# Presence of *Blastocystis* in gut microbiota is associated with cognitive traits and decreased executive function

**DOI:** 10.1038/s41396-022-01262-3

**Published:** 2022-06-21

**Authors:** Jordi Mayneris-Perxachs, María Arnoriaga-Rodríguez, Josep Garre-Olmo, Josep Puig, Rafael Ramos, Maria Trelis, Aurelijus Burokas, Clàudia Coll, Cristina Zapata-Tona, Salvador Pedraza, Vicente Pérez-Brocal, Lluís Ramió, Wifredo Ricart, Andrés Moya, Mariona Jové, Joaquim Sol, Manuel Portero-Otin, Reinald Pamplona, Rafael Maldonado, José Manuel Fernández-Real

**Affiliations:** 1 Department of Diabetes, Endocrinology and Nutrition, Dr. Josep Trueta University Hospital, Girona, Spain; 2 Nutrition, Eumetabolism and Health Group, Girona Biomedical Research Institute (IdibGi), Girona, Spain; 3 CIBER Fisiopatología de la Obesidad y Nutrición (CIBEROBN), Instituto de Salud Carlos III, Madrid, Spain; 4 Serra-Hunter Fellow, Department of Nursing, University of Girona, Girona, Spain; 5 Research Group on Aging, Disability and Health, Girona Biomedical Research Institute (IdibGi), Girona, Spain; 6 Institut Investigació Germans Trias i Pujol (IGTP), Comparative Medicine and Bioimage of Catalonia, Barcelona, Spain; 7 Medical Imaging, Girona Biomedical Research Institute (IdibGi), Girona, Spain; 8 Department of Radiology (IDI), Dr. Josep Trueta University Hospital, Girona, Spain; 9 Vascular Health Research Group of Girona (ISV-Girona). Jordi Gol Institute for Primary Care Research (Institut Universitari per a la Recerca en Atenció Primària Jordi Gol I Gurina -IDIAPJGol), Girona Biomedical Research Institute (IDIBGI), Dr. Josep Trueta University Hospital, Catalonia, Spain; 10 Girona Biomedical Research Institute (IDIBGI), Dr. Josep Trueta University Hospital, Catalonia, Spain; 11 Parasite & Health Research Group, Department of Pharmacy, Pharmaceutical Technology and Parasitology, Faculty of Pharmacy, University of Valencia, Burjassot, 46100 Valencia, Spain; 12 Joint Research Unit on Endocrinology, Nutrition and Clinical Dietetics, University of Valencia-Health Research Institute La Fe, Valencia, Spain; 13 Laboratory of Neuropharmacology, Department of Experimental and Health Sciences, Universitat Pompeu Fabra, Barcelona, Spain; 14 Institute of Biochemistry, Life Sciences Center, Vilnius University, Vilnius, Lithuania; 15 Neuroimmunology and Multiple Sclerosis Unit, Department of Neurology, Dr. Josep Trueta University Hospital, Girona, Spain; 16 Area of Genomics and Health, Foundation for the Promotion of Sanitary and Biomedical Research of Valencia Region (FISABIO-Public Health), Valencia, Spain; 17 Biomedical Research Networking Center for Epidemiology and Public Health (CIBERESP), Madrid, Spain; 18 Institute for Integrative Systems Biology (I2SysBio), University of Valencia and Spanish National Research Council (CSIC), Valencia, Spain; 19 Department of Experimental Medicine, University of Lleida-Lleida Biomedical Research Institute (UdL-IRBLleida), E-25198 Lleida, Spain; 20 Institut Català de la Salut, Atenció Primària, Lleida, Spain; 21 Research Support Unit Lleida, Fundació Institut Universitari per a la recerca a l’Atenció Primària de Salut Jordi Gol i Gurina (IDIAPJGol), Lleida, Spain; 22 Hospital del Mar Medical Research Institute (IMIM), Barcelona, Spain

**Keywords:** Biomarkers, Pathogenesis, Diagnosis

## Abstract

Growing evidence implicates the gut microbiome in cognition. *Blastocystis* is a common gut single-cell eukaryote parasite frequently detected in humans but its potential involvement in human pathophysiology has been poorly characterized. Here we describe how the presence of *Blastocystis* in the gut microbiome was associated with deficits in executive function and altered gut bacterial composition in a discovery (*n* = 114) and replication cohorts (*n* = 942). We also found that *Blastocystis* was linked to bacterial functions related to aromatic amino acids metabolism and folate-mediated pyrimidine and one-carbon metabolism. *Blastocystis*-associated shifts in bacterial functionality translated into the circulating metabolome. Finally, we evaluated the effects of microbiota transplantation. Donor’s *Blastocystis* subtypes led to altered recipient’s mice cognitive function and prefrontal cortex gene expression. In summary, *Blastocystis* warrant further consideration as a novel actor in the gut microbiome-brain axis.

## Introduction

The possible existence of a gut-microbiota brain axis was initially proposed in animal models [1, 2], with increased evidence in humans in recent years [3–5]. It seems that a dialogue between the metagenome and cognitive function exists in parallel to changes in plasma and fecal metabolomics [3–5]. The majority of studies evaluating human gut microbiota in association with phenotypic traits have highlighted the more abundant bacterial components. However, it is well known that homeostasis in the intestine is maintained through communication and interaction between bacteria and a variety of microorganisms, such as eukaryotes [6]. *Blastocystis* is a common gut eukaryote frequently detected in humans capable of asymptomatic long-term host colonisation [7, 8]. The presence of *Blastocystis* has been generally associated with higher gut bacterial diversity, commonly seen in the composition of a healthy microbiota [9–11]. Across 12 metagenomic datasets, a strong association between *Blastocystis* and the enrichment of Firmicutes and Clostridiales, as well as the reduction in Bacteroides has been described [8]. Based on these observations, a commensal role for Blastocystis has been proposed.

The pathogenic potential of Blastocystis has also been reported [12]. For instance, it has been demonstrated that Blastocystis can decrease the abundance of beneficial bacteria [13, 14] and has been associated with irritable bowel disease [15] and inflammatory bowel disease [16]. The negative effects of *Blastocystis* could be mediated not only directly, but also through alterations in the bacterial composition. Hence, two recent studies have revealed that *Blastocystis* caused a decrease in beneficial bacteria, particularly *Bifidobacterium* and *Lactobacillus* [13, 14]. Men with intestinal bowel disease had a significant decrease in *Bifidobacterium* sp. when infected by *Blastocystis* [13]. An in vitro co-incubation assay demonstrated that a bidirectional influence between *Blastocystis* and different bacteria exists: while *Blastocystis* ST7 cell count was higher in the presence of gut bacteria [14], *Blastocystis* ST7 had a selective influence on bacterial specific groups. *Blastocystis* boosted the growth of *Escherichia coli* (a facultative anaerobe) while inhibiting *Bifidobacterium longum* (an obligate anaerobe) and *Lactobacillus* sp. In fact, various epidemiological studies have been conducted to investigate the links between *Blastocystis* and dysbiosis [11].

The commensalism or pathogenicity of *Blastocystis* may be largely dependent on the subtype [11]. Therefore, future analyses should be performed at the subtype level to determine if it is a commensal or a pathogen [11]. There are currently 22 known *Blastocystis* subtypes (ST), but only ST1 to ST9 have been identified in humans, with ST1-4 accounting for the vast majority of strains (>90%) [17]. We aimed to evaluate whether the presence of *Blastocystis* in a discovery (*n* = 114) and replication cohorts (*n* = 942) was associated to cognitive traits through changes in the gut microbiota composition and functionality reflected in changes in metabolomics of plasma. To validate these observations, we finally transplanted the microbiota to mice to test possible behavioural effects.

## Methods

### Clinical study

#### Longitudinal discovery cohort (IRONMET, baseline: *n* = 114, 1-year follow-up: *n* = 75)

Eligible subjects were evaluated in the Endocrinology Department of Dr. Josep Trueta University Hospital. We included consecutive subjects with obesity (body mass index, BMI ≥ 30 kg/m^2^) and age- and sex-matched non-obese subjects BMI 18.5 − 30 kg/m^2^. Exclusion criteria were: type 2 diabetes mellitus, chronic inflammatory systemic diseases, acute or chronic infections in the previous month; use of antibiotic, antifungal, antiviral or treatment with proton-pump inhibitors; severe disorders of eating behaviour or major psychiatric antecedents; neurological diseases, history of trauma or injured brain, language disorders; and excessive alcohol intake (≥40 g OH/day in women or 80 g OH/day in men). Cognitive tests were collected again in 75 consecutive subjects after 1 year of follow-up. The Institutional review board—Ethics Committee and the Committee for Clinical Research (CEIC) of Dr. Josep Trueta University Hospital (Girona, Spain) approved the study protocol and informed written consent was obtained from all participants.

#### Validation cohort 1 (IMAGEOMICS, *n* = 942)

The Aging Imageomics Study is an observational study including participants from two independent cohort studies (MESGI50 and MARK). Detailed description of the cohorts can be found elsewhere [18]. Briefly, the MESGI50 cohort included a population aged ≥50 years old, while the MARK cohort included a random sample of patients aged 35–74 years with intermediate cardiovascular risk. Eligibility criteria included age ≥50 years, dwelling in the community, no history of infection during the last 15 days, no contraindications for MRI, and consent to be informed of potential incidental findings. The Ethics committee of the Dr. Josep Trueta University Hospital approved the Aging Imageomics Study protocol.

#### Validation cohorts 2 and 3 (*n* = 31 and *n* = 19)

These are independent cohorts with the same inclusion and exclusion criteria as the discovery cohort. The Institutional review board—Ethics Committee and the Committee for Clinical Research (CEIC) of Dr. Josep Trueta University Hospital (Girona, Spain) approved the study protocol and informed written consent was obtained from all participants.

### Neuropsychological assessment in humans

#### Total Digit Span (TDS)

Working memory was assessed by the Digit Span, a subtest of the Wechsler Adult Intelligence Scale-III (WAIS-III) [19] a measure of general intellectual function. It is based on numbers and includes the Forward and Backward Digit Span tests. In the Forward Digit Span test, the examinee repeats a number sequence in the same order as presented. This constitutes a measure of working memory but also of attention. In the Backward Digit Span, the examinee repeats the number sequence in reverse order. Total Digit Span represents the total score of the two previous tests. A higher score reflects a better memory function. In a standardization sample of 394 participants (aged 16–89 years), the reliability coefficient was relatively high, ranging from 0.94–0.97 [20].

#### Trail Making Test

The Trail Making Test (TMT) is composed by the subtests TMTA and TMTB. The TMTA (TMTA, greater focus on attention) consisted of a standardized page in which numbers 1 to 25 are scattered within the circles, and participants were asked to connect the numbers in order as quickly as possible. Before starting the test, a 6-item practice test was administered to the participants to make sure they understood both tasks. A maximum time of 300 s was allowed before suspending the test. The direct scores of TMTA were the time in seconds taken to complete each task. In the same way, TMTB (Trail B, greater focus on executive function) consisted of an alternating sequence of numbered circles and letters [21, 22]. In both tests, shorter times to completion indicate better performance.

#### Stroop Color-Word Test

The Stroop Color-Word Test (SCWT) (Golden’s version) was administered to assess cognitive flexibility, selective attention, inhibition and information processing speed. This version consists of three different parts: (1) 100 words (color names) are printed in black ink and the subject is asked to read them as fast as possible, (2) 100 “XXX” are printed in color ink (green, blue and red) and the subject is asked to name as fast as possible the ink color, and (3) 100 color names (from the first page) printed in color ink (from the second page), the color name and the ink color do not match and the subject is asked to name the ink color (and not to read the color name). The subject is given 45 seconds for each task, after the 45 s the last item completed is noted, obtaining three scores: one for each part of the test (“P”, “C” and “PC”). The interference (“I”) index was also obtained from the subtraction PC-PC’ (PC’ = P x C/P + C). Standard administration procedures were followed as indicated in the test manual [23].

#### Phonemic Verbal Fluency

The Phonemic Verbal Fluency (PVF) is a spontaneous verbal production task that consists to produce words with a specified letter (P) during one minute. This task is a measure of language ability and executive function and is influenced by processing speed. The number of correct words was scored and higher numbers indicate better performance.

### Extraction of faecal genomic DNA and whole-genome shotgun sequencing

#### IRONMET (discovery cohort) and validation cohort 3

Total DNA was extracted from frozen human stool using the QIAamp DNA mini stool kit (QIAGEN, Courtaboeuf, France) following the manufacturer’s instructions. Quantification of DNA was performed with a Qubit 3.0 fluorometer (Thermo Fisher Scientific, Carlsbad, CA, USA), and 1 ng of each sample (0.2 ng/µl) was used for shotgun library preparation for high-throughput sequencing, using the Nextera DNA Flex Library Prep kit (Illumina, Inc., San Diego, CA, USA) according to the manufacturer’s protocol. Sequencing was carried out on a NextSeq 500 sequencing system (Illumina) with 2 × 150-bp paired-end chemistry, at the facilities of the Sequencing and Bioinformatic Service of the FISABIO (Valencia, Spain). The obtained input FASTq files were decompressed, filtered and 3' ends-trimmed by quality, using prinseq-lite-0.20.4 program [24] and overlapping pairs were joined using FLASH-1.2.11 [25]. Fastq files, converted into fasta files, were mapped against the reference human genomes (GRCh38.p11, Dec 2013 and GRCm38.p6, Sept 2017), respectively, to remove reads from host origin, by using bowtie2-2.3.4.3 [26] with end-to-end and very sensitive options. Next, functional analyses were carried out by assembling the host-free reads into contigs by MEGAHIT v1.1.2 [27] and mapping those against the contigs with bowtie2. Reads that did not assemble were appended to the contigs. Then, prediction of codifying regions was implemented by Prodigal v2.6.342 [28], and subsequent functional annotation was carried out with HMMER [29] against the Kyoto Encyclopaedia of Genes and Genomes (KEGG) database, version 2016 [30] to obtain the gene functional annotation. The best annotations were filtered and orf annotation were assigned to every read using the statistical package R 3.1.0 [31] which also was used to count the aligned reads, to add the category and its coverage, and finally to build abundance matrices. Taxonomic annotation was implemented with Kaiju v1.6.2 [32] on the host-free reads, using a greedy mode. Addition of lineage information, counting of taxa and generation of an abundance matrix for all samples were performed using the package R. Finally, non-viral taxa were excluded for the downstream analyses. The presence of the parasite was confirmed by PCR amplification and sequencing of the small subunit (SSU) rRNA gene of the parasite.

#### IMAGEOMICS (validation cohort 1) and validation cohort 2

DNA was extracted from stool samples using the PowerSoil DNA extraction kit (MO BIO Laboratories) following the manufacturer’s protocol. Between 400 and 500 ng of total DNA were used for library preparation for Illumina sequencing employing DNA Prep kit (Illumina). All libraries were assessed using a TapeStation High Sensitivity DNA kit (Agilent Technologies) and quantified by Qubit (Invitrogen). Validated libraries were pooled in equimolar quantities and sequenced as a paired-end 150-cycle run on an Illumina NextSeq2000. Raw reads were filtered for QV > 30 using an in-house phyton script.

For the taxonomic and functional diversity analysis of the microbiota present in the samples, FASTq output files were first preprocessed using fastp [33], a FASTq data pre-processing tool for quality control, trimming of adapters, and quality filtering. Clean reads were mapped against the *Homo sapiens* genome database (GRCh38.p13) using Bowtie2 [26] to remove reads from human origin. Unmapped reads were run using the SqueezeMeta v 1.3.1 [34] using the co-assembly mode to pool all samples in a single assembly. Contigs assembly was carried out with megahit [27] or mapping reads in contigs bowtie2 was the choice, Prodigal [28] was used for ORFs prediction, Diamon [35] for ORF search and alignment against the GenBank nr database for taxonomic assignment, and KEGG database for functional annotation.

The resulting functional and taxonomic tables of abundances in the discovery (IRONMET) and validation (IMAGEOMICS) cohorts served for the search of *Blastocystis* hits in the samples. In order to identify/verify the particular subtype of *Blastocystis* matching the positive hits identified at species level by Squeezmeta, blast searches were carried out using all contigs from the program Squeezmeta that had previously matched with *Blastocystis* sp. as the query ones for blastn sequences against an in-house database built from 10 available genomes from isolates/strains of *Blastocystis* detected humans (downloaded from https://www.ncbi.nlm.nih.gov/datasets/genomes/?taxon=blastocystis) spanning subtypes 1(Nand II), 2 (Flemming), 3 (ASY-1 and ZGR), 4 (BT1 and WR1), 6 (SSI:754), 7 (isolate B), 8 (Dmp/08-128), and 9 (F5323). Only hits with at a minimum of 70% identity and over 70% of the contig length were considered as being potentially of *Blastocystis* origin for subsequent subtype assignment. The first hit for each query was selected based on its bitscore as the identified subtype and reads counts sorted by subtype were obtained for each sample, and finally an abundance table of the different subtypes of *Blastocystis* was created for all samples.

Metabolomics Analyses, Faecal Microbiota Transplantation, Study of Gene Expression in mouse Prefrontal Cortex, Metagenomics statistical analysis Metabolomics statistical analysis and RNA-seq analysis are described in Supplementary files.

### *Blastocystis* centred log ratio values

Taxa and bacterial functions were previously filtered so that only those with more than 10 reads in at least 10% of the samples were selected. Then, to take into account the compositional structure of the microbiome data and rule out possible spurious associations, we applied a centred log-ratio (*clr*) transformation to the filtered raw counts for each sample (*j*):

$$clr({{{{{{{\boldsymbol{N}}}}}}}}_{{{{{{{\boldsymbol{j}}}}}}}}) = \left[ {log\left( {\frac{{n_{1j}}}{{g({{{{{{{\boldsymbol{N}}}}}}}}_{{{{{{{\boldsymbol{j}}}}}}}})}}} \right), \ldots ,log\left( {\frac{{n_{Dj}}}{{g({{{{{{{\boldsymbol{N}}}}}}}}_{{{{{{{\boldsymbol{j}}}}}}}})}}} \right)} \right]$$

where n_*ij*_ are the raw counts for the *i*th taxon in the *j*th sample and g*(****N****)* is the geometric mean of ***N***, $g({{{{{{{\boldsymbol{N}}}}}}}}) = \root {D} \of {{n_1n_2 \ldots n_D}}$. The geometric mean cannot be determined for sparse data without replacing or estimating the 0 counts. They were estimated as a probability distribution using the “ALDEx2” R package [36]. It uses a Bayesian approach using the actual data as a prior to calculate a posterior distribution of the data. Thus, the table of reads counts for each feature was converted to a distribution of posterior probabilities using a Monte Carlo sampling (K = 128 instances) from a Dirichlet distribution for each sample: $[p_1,p_2, \ldots ]\sim Dirichlet\left( {\left[ {n_1,n_2, \ldots } \right] + \frac{1}{2}} \right)$. An uninformative prior of ½ was used to model the frequency of features with zero counts. This multivariate distribution ensures that none of the inferred proportions is ever exactly zero even if the associated count is zero, and that the probability is conserved. The marginal distributions of *p*_i_ are wide when the associated read count is small and narrow if the number of counts are large. Therefore, it accounts for the fact that one read out of 100 total reads has much lower precision than 1000 reads out of 100,000 total reads, despite having the same proportion. Each Monte Carlo instance *p*_kj_ was then transformed using a *clr* transformation. Finally, we calculated the median of the *clr*-transformed instances and obtained the corresponding values for the *Blastocystis* subtypes. The *clr*-transformed values were then used either as a continuous variable in linear models or Spearman correlations to assess associations with the scores of cognitive tests, bacterial species/KEGG orthologues or metabolites.

## Results

### *Blastocystis* subtypes are associated with deficits in executive function and altered gut bacterial composition

We first assessed the relationship of *Blastocystis* subtypes 1 (ST1), 2 (ST2), 3 (ST3), and 4 (ST4) with executive function in a longitudinal discovery cohort (IRONMET, *n* = 114, Table S1). The mean relative abundance and prevalence (≥5 counts) of each subtype (ST1, ST2, ST3, ST4) in this cohort based on shotgun metagenomics data were 0.0045, 0.00016, 0.00013, 0.034, and 25.4%, 6.1%, 6.1%, 23.7%, respectively (Table S2), and 81 patients (71%) had <5 counts for all of these four subtypes (non-carriers). We confirmed these results by RT-PCR. Thus, we found a sensitivity of 84.4% and a specificity of 93.4% when compared with the RT-PCR results (Table S2). Among *Blastocystis* carriers, 30% harboured a single subtype (6 ST1 and 4 ST4) whereas 70% contained mixed populations (12 ST1+ST4; 4 ST1+ST3+ST4; 6 ST1+ST2+ST4; and 1 with all four subtypes) (Table S2). The correlation between both methods was 0.78 (*p* < 0.001) assessed by the Cramer’s V.

**Fig. 1:**
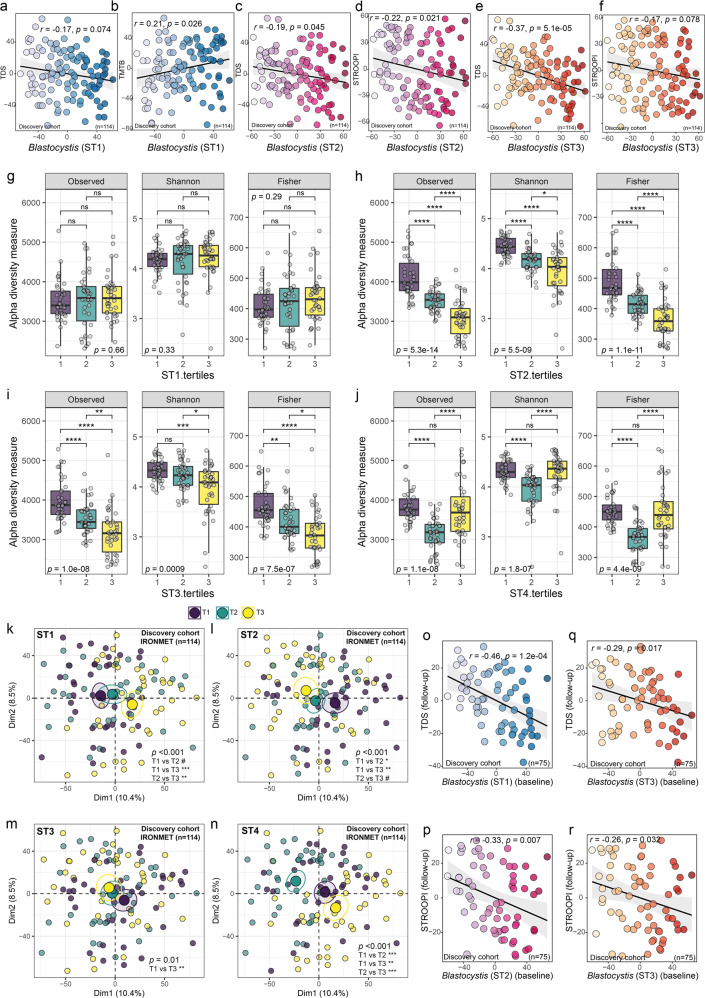
*Blastocystis* subtypes are associated with executive function and microbial diversity in a longitudinal discovery cohort. **a**–**f** Scatter plots of the partial Spearman’s rank correlations (adjusted for age, BMI, sex and education years) between the baseline faecal centered log-ratio (clr) transformed *Blastocystis* subtypes values and executive function assessed by the total digit span test (TDS), the trail making test part-B (TMTB) and the Stroop interference (STROOPI) in the discovery cohort (IRONMET, *n* = 114) at baseline. The ranked residuals are plotted. **g** Boxplots of alpha diversity indices according to the tertiles of the clr-transformed *Blastocystis* subtype 1 (ST1), **h** subtype 2 (ST2), **i** subtype 3 (ST3), and **j** subtype 4 (ST4). Global differences were assessed using a Kruskal-Wallis test and pairwise comparisons were assessed using a Wilcoxon rank sum tests with Bonferroni multiple testing correction. **k** Principal component analysis scores plot of the clr-transformed microbial data coloured according to the clr-transformed ST1 tertiles, **l** ST2 tertiles, **m** ST3 tertiles, and **n** ST4 tertiles. Overall differences in the microbiome composition were assessed by PERMANOVA using 1000 permutations and Euclidean distances. Pairwise differences between groups were assessed using the pairwise.adonis function adjusted for Bonferroni correction. ^#^*p* < 0.1; *p < 0.05; **p < 0.01, ***p < 0.001. **o**–**r** Scatter plots of the partial Spearman’s rank correlations (adjusted for age, BMI, sex and education years) between the baseline faecal centered log-ratio (clr) transformed *Blastocystis* subtypes values and executive function assessed by the TDS and the STROOPI in the discovery cohort after 1-year of follow-up (IRONMET, *n* = 75).

After applying the *clr* transformation to take into account the compositional nature of the microbiome data, all *Blastocystis* subtypes were negatively associated with cognitive tests specific for executive function (Fig. 1a–f). In particular, ST1 was associated with the digit span test scores (TDS) and the part B of the trail making test (TMTB), while ST2 and ST3 were related to deficits in executive function through the TDS and the Stroop Interference tests (STROOPI). Finally, ST4 was also negatively associated with the backward digit span test (Fig. S1a). These associations were replicated: *Blastocystis* levels at baseline were negatively associated with executive functioning one year later in a subset of participants of the discovery cohort (*n* = 75) (Fig. 1o–r).

The presence of *Blastocystis* has been generally associated with higher gut bacterial diversity [9–11]. However, we found no associations among alpha diversity indices (observed richness, Shannon, and Fisher) and the tertiles of the *clr*-transformed ST1 subtypes (Fig. 1g), although there seemed to be a trend towards increased diversity with higher tertiles. In addition, higher values of both ST2 and ST3 were associated with lower diversity in all measures (Fig. 1h, i). In the case of ST4, both the lowest and highest tertiles were associated with the highest alpha diversity, although there were no differences between them (Fig. 1j).

Recent studies have suggested that the effects of *Blastocystis* on the host could be mediated not only directly but also through modulation of the gut microbiota composition [9–11, 14, 37]. The compositional alternative to the principal coordinate analysis plots of β-diversity is the principal component analysis (PCA). Therefore, we applied a PCA analysis to the *clr*-transformed microbial data to reveal global variance patterns in the microbial profiles according to the *Blastocystis* subtypes. This initial unsupervised exploratory analyses revealed significant differences in the bacterial composition according to all *clr*-transformed *Blastocystis* subtypes, with ST4 and ST3 having the highest and lowest differences among tertiles (Fig. 1k–n).

**Fig. 2:**
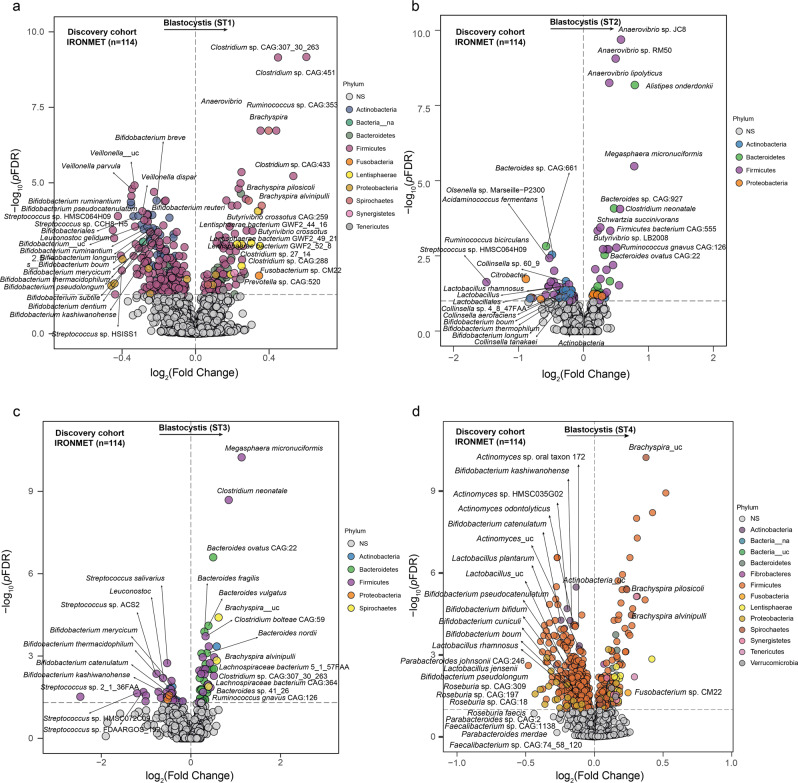
*Blastocystis* subtypes are associated with bacterial composition in a longitudinal discovery cohort. **a** Volcano plots of differential bacterial abundance associated with the faecal *clr*-transformed *Blastocystis* subtype 1 (ST1), **b** subtype 2 (ST2), **c** subtype 3 (ST3), and **d** subtype 4 (ST4) values in the discovery cohort identified with DESeq2 using the geometric mean of pairwise ratios normalization to specifically account for the compositional and zero-inflated microbiome data and controlling for age, sex, BMI, and education years. The log2 fold change associated with a unit change in the *clr*-transformed values and the log10 *p* values adjusted for multiple testing (pFDR) are plotted for each taxon. Significantly different taxa are coloured according to phylum.

We next evaluated the associations of the different *Blastocystis* subtypes with bacterial composition using generalized linear models (controlling for age, sex, BMI, and years of education) with DESeq2 using the geometric mean of pairwise ratios (GMPR) normalization [38] to specifically account for the compositional and zero-inflated properties of the microbiome data. *Blastocystis* ST1 was strongly negatively associated with bacterial species belonging to the Actinobacteria phylum, particularly from the *Actinomyces, Bifidobacterium* and *Collinsella* genera (Fig. 2a, Table S3), species from the Lachnospiraceae family (*Blautia* spp., *Roseburia* spp.) and *Faecalibacterium* genus (*Faecalibacterium prausnitzii*), which are known short chain fatty acid (SCFA)-producers, and *Lactobacillus* species, and *Parabacteroides* species. Conversely, species from the Lentisphaerae phylum and from the *Prevotella*, *Bacteroides, Brachyspira*, and *Fusobacterium* genera run in parallel to *Blastocystis* ST1. Similarly, ST2 was negatively associated with several *Bifidobacterium, Collinsella, Actinomyces* and *Lactobacillus*, but positively with *Bacteroides and Prevotella* species (Fig. 2b, Table S4). In the case of ST3, we also found consistent negative associations with *Bifidobacterium and Actinomyces* spp. and positive associations with species from the *Bacteroides, Fusobacterium*, and *Brachyspira* genera (Fig. 2c, Table S5). Similarly, ST4 was strongly negatively associated with species from the Actinobacteria phylum (*Bifidobacterium, Actinomyces, Collinsella*), species from the Lachnospiraceae family (*Blautia, Dorea, Roseburia*), and species from the *Faecalibacterium, Lactobacillus* and *Parabacteroides* genera (Fig. 2d, Table S6). We further analysed the bacterial associations with the *Blastocystis* subtypes considering an alternative approach based on applying linear models as implemented in the “limma” R package [39] to the *clr*-transformed bacterial data. Again, we found that the *clr*-transformed levels of the different *Blastocystis* subtypes were strongly negatively associated with *Bifidobacterium, Collinsella, Actinomyces, Lactobacillus* and SCFA-producing species, but positively with species from the *Brachyspira* genera (Fig. S2a–c and Tables S7–S10).

### *Blastocystis* subtypes are associated with bacterial functions related to pyrimidine, one-carbon, and aromatic amino acids metabolism

**Fig. 3:**
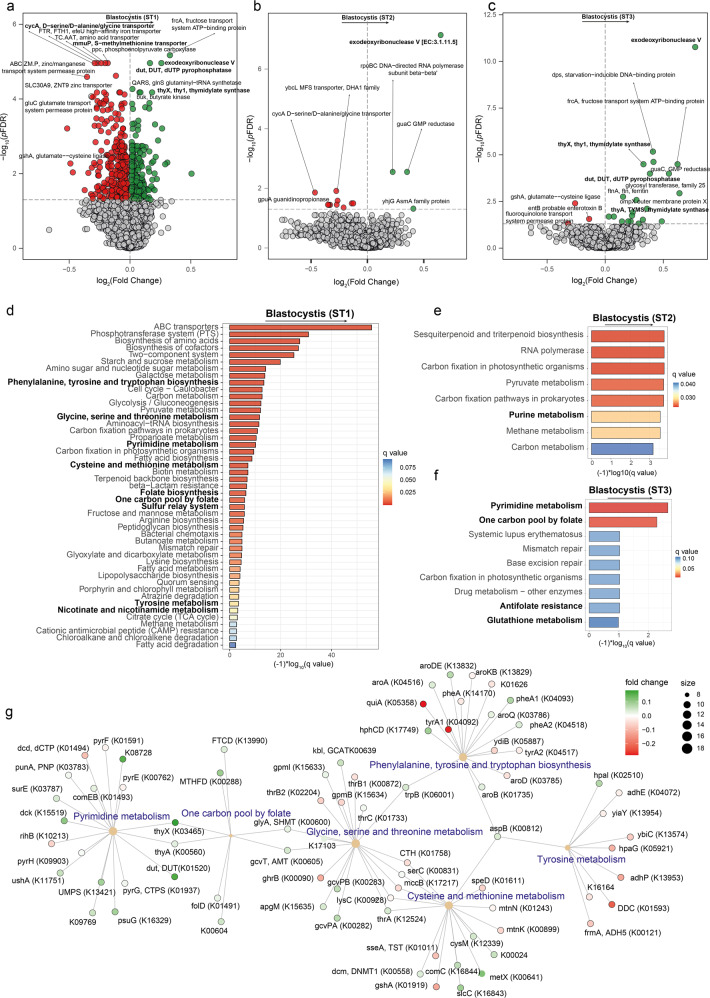
*Blastocystis* subtypes are linked to bacterial functionality related to aromatic amino acid metabolism and folate-mediated pyrimidine and one-carbon metabolism. **a** Volcano plots of bacterial functions associated with the faecal clr-transformed *Blastocystis* subtype 1 (ST1), **b** subtype 2 (ST2), and **c**) subtype 3 (ST3) values in the discovery cohort (IRONMET, *n* = 114) identified with DESeq2 using the geometric mean of pairwise ratios normalization to specifically account for the compositional and zero-inflated microbiome data and controlling for age, sex, BMI, and education years. The log2 fold change associated with a unit change in the *clr*-transformed values and the log10 p-values adjusted for multiple testing (pFDR) are plotted for each function. Significantly different taxa are coloured in green (upregulated) or red (down-regulated). **d**–**f** Manhattan-like plot of the KEGG pathway over-representation analyses (*q* value < 0.1) mapping the KEGG orthologues significantly associated with *Blastocystis* ST1, ST2, and ST3, respectively. Bars are coloured according to the *q* value. **g** Gene-concept network depicting the linkage of significant KEGG orthologues participating in KEGG pathways related to the aromatic amino acids metabolism and folate-mediated pyrimidine and one-carbon metabolism for *Blastocystis* ST1.

We also evaluated the possible associations with the microbiome functionality. Reads originating from microbial genes were mapped to the Kyoto Encyclopaedia of Genes and Genomes (KEGG) orthologues and used to identify microbiome functions associated with the different *Blastocystis* subtypes controlling for age, sex, BMI and years of education (Fig. 3a–c, Tables S11–14). In ST1, ST2 and ST3 we identified a strong positive association with microbial functions involved in the biosynthesis of purines and pyrimidine metabolism through the folate-mediated one-carbon metabolism, particularly exodeoxyribonuclease V, thymidylate synthase (*thyX, thyA)* and dUTP pyrophosphatase (*dut*). We also found strong associations with other microbial functions linked to metabolites participating in the one-carbon metabolism such as serine, glycine or cysteine. These microbial functions were not significantly associated with ST4, which could partially explain the fact that this subtype was less associated with measurements of executive functioning compared with ST1-3.

To gain further insights, we next performed pathway over-representation analyses mapping significant KEGG orthologues to the KEGG pathways (Fig. 3d–f, Fig. S1b). This enrichment analyses further highlighted a significant (*q* value<0.1) over-representation of pathways associated with purine and pyrimidine metabolism and one-carbon metabolism, particularly in ST1 and ST3, as well as pathways fuelling one-carbon sources such as glycine, serine, threonine, cysteine, methionine, and folate. In addition, we also identified pathways involved in aromatic amino acids metabolism, which we have previously shown that play an important role in executive function [5]. These analyses highlighted other important functions (in addition to *thyX, thyA*, and *dut*) with an important role in the folate-mediated one-carbon metabolism such as formiminotetrahydrofolate cyclodeaminase (*FTCD*), methylenetetrahydrofolate dehydrogenase (*MTHFD*), formyltetrahydrofolate synthetase (*folD*), glycine hydroxymethyltransferase (*glyA, SHMT*), and aminomethyltransferase (*gcvT, AMT*) (Figs. 3g and S1c). Many of these pathways have been linked to executive function in a recent study [5].

To further validate these results, we also analysed the associations of the *Blastocystis* subtypes with microbial functionality applying linear models (as implemented in the “limma” R package [39]) to the clr-transformed KEGG orthology data. Again, we found a strong consistency with the results obtained using DESeq2 and the GMPR normalization, with an overlap in the identified associated orthologs between the two approaches ranging from 50% for the ST3 to 92% for ST2 (Tables S15–S18). Furthermore, both *thyA* and *dut* were only associated with all subtypes except for ST4 (Tables S15–S19). Sixteen KEGG orthologues were associated with all *Blastocysits* subtypes (Fig. S2d). As a result, we also found a strong consistency in the over-represented KEGG pathways associated with the *Blastocystis* subtypes (Fig. S2e–h).

We sought to validate these findings in subjects from an independent large-scale validation cohort (IMAGEOMICS, *n* = 942, Table S20) who underwent a battery of cognitive tests. The mean relative abundance and prevalence of each subtype (ST1, ST2, ST3, ST4) in this cohort were 0.035, 0.00073, 0.0069, 0.0038, and 43.8%, 28.1%, 28.1%, 30.0%, respectively (Table S2), and 392 patients (41.6%) had no counts for any of these for subtypes (non-carriers). Among *Blastocystis* carriers, 42.7% had a single subtype, whereas 57.3% harboured a mixed population (Table S2). *Blastocystis* may be a marker for the presence of other single-cell eukaryote. However, although we found other eukaryote to be present in the samples (Table S2), *Blastocystis* prevalence and mean relative abundances were the highest by far.

**Fig. 4:**
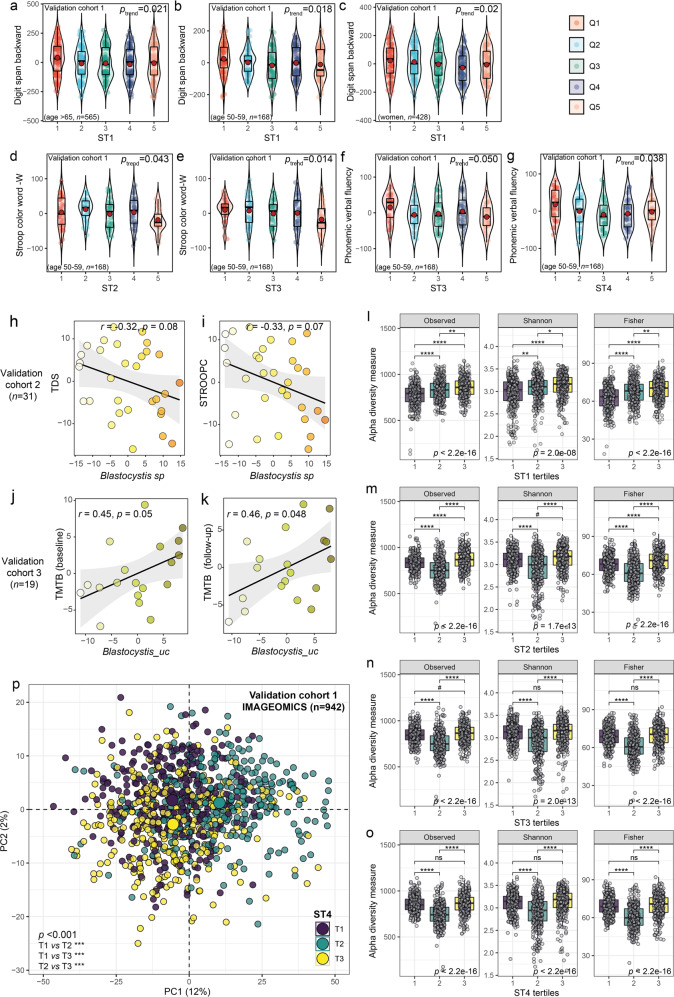
*Blastocystis* subtypes are associated with executive function in three independent validation cohorts. Violin plots for different cognitive tests according to the quintiles of the *Blastocystis* subtypes in a subset of subjects from the validation cohort 1 (IMAGEOMICS): **a**–**c** the Digit Span Backward for the *Blastocystis* subtype 1 (ST1) in subjects aged ≥ 65 (*n* = 565), subjects aged 50–59 (*n* = 168), women (*n* = 428), respectively, (**d**) the Stroop Colour Word – word test for the *Blastocystis* subtype 2 (ST2) in subjects aged 50–59 (*n* = 168), **e**, **f** the Stroop Colour Wordword and Phonemic Verbal Fluency for the *Blastocystis* subtype 3 (ST3) in subjects aged 50–59 (*n* = 168), and (**g**) Phonemic Verbal Fluency for the *Blastocystis* subtype 4 (ST4) in subjects aged 50–59 (*n* = 168). **h** Scatter plots of the partial Spearman’s rank correlations (adjusted for age, BMI, sex and education years) between the baseline faecal centered log-ratio (clr) transformed *Blastocystis* sp. values and executive function assessed by the total digit span test (TDS) and (**i**) the trail making test part-B (TMTB) in the validation cohort 2 (*n* = 31), and **j** the clr-transformed *Blastocystis* levels and TMTB in the validation cohort 3 (*n* = 19) at baseline and (**k**) after 3-year follow-up. **l** Boxplots of alpha diversity indices according to the tertiles of the clr-transformed ST1, (**m**) ST2, (**n**) ST3, and (**o**) ST4 in the validation cohort 1 (IMAGEOMICS, *n* = 942). Global differences were assessed using a Kruskal–Wallis test and pairwise comparisons were assessed using a Wilcoxon rank sum tests with Bonferroni multiple testing correction. The ranked residuals are plotted. **p** Principal component analysis scores plot of the clr-transformed microbial data coloured according to the clr-transformed ST4 tertiles in the validation cohort 1 (IMAGEOMICS, *n* = 942). Overall differences in the microbiome composition were assessed by PERMANOVA using 1000 permutations and Euclidean distances. Pairwise differences between groups were assessed using the pairwise.adonis function adjusted for Bonferroni correction. ^#^*P* < 0.1, *P < 0.05, **P < 0.01, ***P < 0.001.

In all subjects as a whole, we did not observe significant associations between the clr-transformed *Blastocystis* sp. and any of the cognitive tests assessing specifically executive functioning after controlling for age, sex, BMI and years of education. However, we must take into account that participants in the IMAGEOMICS cohort were considerably older (age range 50–98 years) than those from the IRONMET cohort (age range: 27–65) and age has been described as the most important covariate associated with *Blastocystis* carrier status [10]. In addition, patients in the discovery cohort were mainly women (80%, Table S1), whereas only 46% of participants in the validation cohort were women (Table S20). Considering different age groups, we found ST1 were negatively associated with the digit backward span both in subjects ≥65 years (Fig. 4a) and <60 years (Fig. 4b). In the case of women (*n* = 428), we also found deficits in executive function associated with ST1 independently of age (Fig. 4c). For the other *Blastocystis* subtypes, we only found significant associations with executive function in participants <60 years old (Fig. 4d–g). As sanitation and socioeconomic status may explain transmission of *Blastocystis* among humans, we further controlled the associations with the cognitive tests for the total income of the participants. Consistent with the previous results, we found significant associations of all subtypes with measures of executive function after controlling for the socioeconomic status (Fig. S3l).

We also assessed the associations of *Blastocystis* with executive function in two smaller cohorts with similar characteristics and inclusion and exclusion criteria that those from the discovery cohort (Tables S21–22). In the second validation cohort (*n* = 31), clr-transformed *Blastocystis* sp. were negatively associated with measures specific for executive function, namely the TDS and the STROOPC tests (Fig. 4h, i). Similarly, in the third validation cohort (*n* = 19), *Blastocystis_uc* were also associated with an impairment of executive function assessed by the TMTB tests, both at baseline (Fig. 4j) after a 3-year follow-up (Fig. 4k).

In addition to validating the associations between *Blastocystis* and impaired executive functioning, we also confirmed the shifts in the gut bacterial composition and functionality. We found an increased in alpha diversity according to the clr-transformed ST1 tertiles (Fig. 4l). In the case of ST2-4, both the lowest and highest tertiles were associated with the highest alpha diversity, although there were no differences between them (Fig. 4m–o), which is consistent with the results observed in the discovery cohort for ST4 (Fig. 1j). A PCA also showed significant differences in the bacterial composition according to all clr-transformed *Blastocystis* subtypes (Fig. 4p, Fig. S3a–c).

**Fig. 5:**
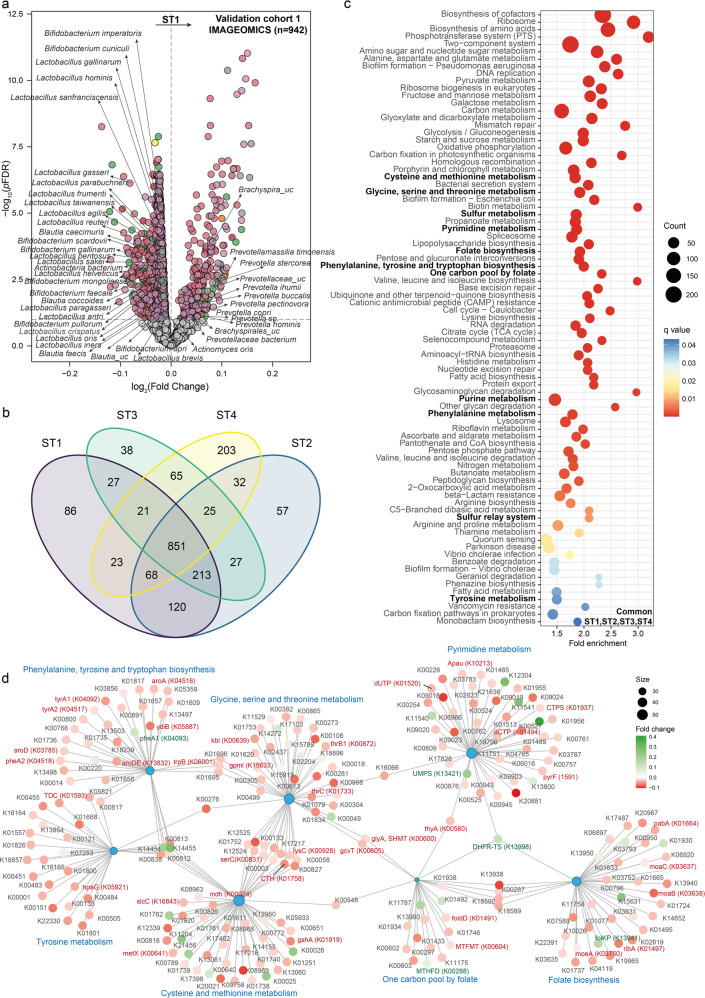
*Blastocystis* are associated with bacterial composition and functionality in a large validation cohort. **a** Volcano plots of bacterial taxa associated with the faecal clr-transformed *Blastocystis* subtype 1 (ST1) values in the validation cohort 1 (IMAGEOMICS, *n* = 942) identified applying linear models to the clr-transformed bacterial data controlling for age, sex, BMI, and education years. The log2 fold change associated with a unit change in the *clr-*transformed values and the log10 *p* values adjusted for multiple testing (pFDR) are plotted for each taxon. Significantly different taxa are coloured according to phylum. **b** Venn diagram representing the overlap of taxa significantly associated with the different *Blastocystis* subtypes in the validation cohort 1 (IMAGEOMICS, *n* = 942). **c** Dot plot of the KEGG pathway over-representation analysis (*q* value < 0.1) mapping the KEGG orthologues significantly associated with all *Blastocystis* subtypes in the validation cohort 1 (IMAGEOMICS, *n* = 942). Dots are coloured according to the *q* value. **d** Gene-concept network depicting the linkage of significant KEGG orthologues associated with all *Blastocystis* subtypes participating in KEGG pathways related to the aromatic amino acids metabolism and one-carbon metabolism.

Consistent with the findings in the discovery cohort, all the clr-transformed *Blastocystis* subtypes levels were strongly negatively associated with clr-transformed species from the Actinobacteria phylum (particularly *Bifidobacterium*), the Lachnospiraceae family (particularly *Blautia*) and the *Lactobacillus* genera, but positively with species from the *Brachyspira* and *Prevotella* genera (Fig. 5a, Fig. S3d–f, Tables S23–26). We found a strong agreement among the bacterial species associated with the *Blastocystis* subtypes in the IMAGEOMICS cohort, with 851 taxa associated with all subtypes (Fig. 5b, Table S27). At the functional level, we also found a strong coincidence in the KEGG orthologues (Tables S28–S31) and KEGG pathways (Fig. S3g–j) associated with all *Blastocystis* subtypes, with 84 common pathways associated to all *Blastocystis* subtypes (Fig. S3k). Enrichment analysis of KEGG microbial functions associated with all *Blastocystis* subtypes highlighted again an over-representation of microbial functions involved in pyrimidine and purine metabolism, and pathways involved in one-carbon metabolism, including folate cycle and biosynthesis, glycine, serine and threonine metabolism, sulphur metabolism and sulphur relay system (Fig. 5c, Table S32). We also found consistent associations with aromatic amino acids biosynthesis and tyrosine and phenylalanine metabolism. Among the significant bacterial functions participating in these pathways in the IMAGEOMICS validation cohort, we found strong coincidence with bacterial functions from the pyrimidine metabolism, folate cycle, and glycine, serine and threonine metabolism involved in the one-carbon metabolism identified in the IRONMET discovery cohort. Thus, we also identified *folD, MTHFD, MTFMT*, thymidylate synthase (*thyA* and DHFR-TS), *dut, glyA* and *gcvT* (*q* value < 0.05, Fig. 5d) in the validation cohort.

### *Blastocystis*-associated shifts in bacterial functionality translate into the circulating metabolome

**Fig. 6:**
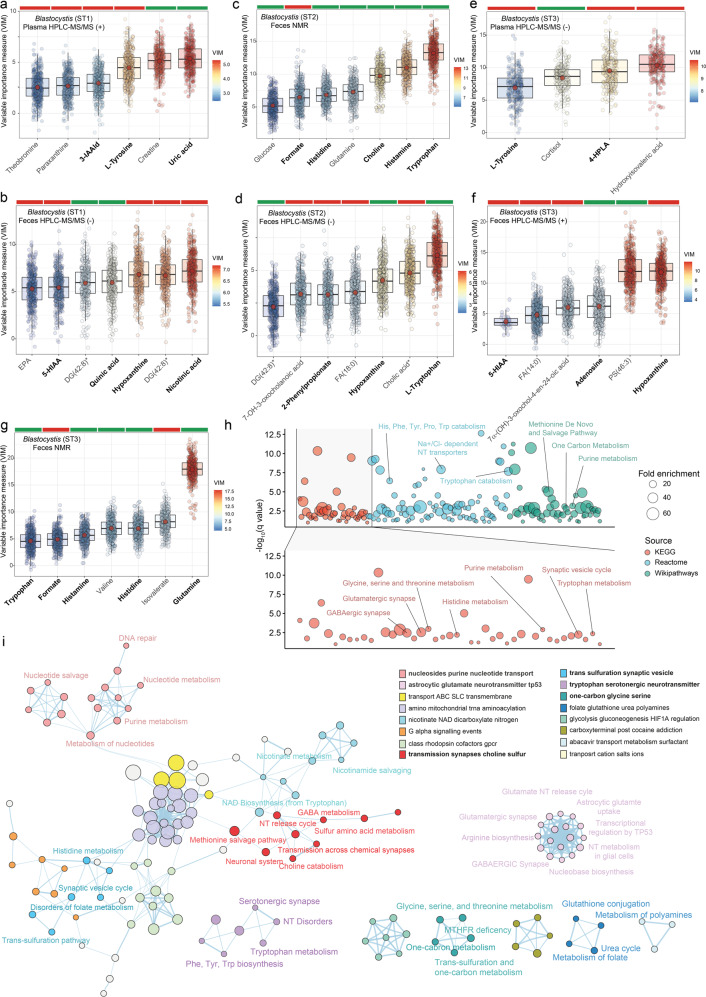
Plasma and faecal metabolites associated with the *Blastocystis* subtypes in the discovery cohort identified by machine learning. Boxplots of the normalized variable importance measure (VIM) for the metabolites associated with the clr-transformed **a** *Blastocystis* ST1 in plasma by HPLC-ESI-MS/MS in positive mode, **b** *Blastocystis* ST1 in faeces by HPCL-ESI-MS/MS in negative model, **c** *Blastocystis* ST2 in faeces by NMR, **d** *Blastocystis* ST2 in faeces by HPLC-ESI-MS/MS in negative mode, **e** *Blastocystis* ST3 in plasma by HPLC-ESI-MS/MS in negative mode, **f** *Blastocystis* ST3 in faeces by HPLC-ESI-MS/MS in positive mode, and **g** *Blastocystis* ST3 in faeces by NMR. The red dot represents the mean and the colour bar above each plot indicates the sign of the association among the corresponding metabolites the *clr*-transformed *Blastocystis* values, with red indicating negative correlation and green positive correlation. Significant metabolites were identified using a multiple random forest-based machine learning variable selection strategy as implemented in the Boruta algorithm with 5000 trees and 500 iterations. For HPLC-MS, all metabolites were identified based on exact mass, retention time and MS/MS spectrum, except those with (*) that were only identified based on exact mass and retention time. **h** Manhattan plot of pathway over-representation analysis (*q* value < 0.1) from metabolites significantly associated with the *Blastocystis* subtypes based on KEGG, Reactome, and Wikipathways databases. The bubble size represents fold enrichment. **i** Over-representation analysis results were mapped as a functional network of pathways using Cytoscape and enrichment map. Nodes represent over-represented pathways. Node size reflects the total number of genes in each pathway. Edge thickness represents the degree of overlapping genes between pathways. Groups of functionally related pathways are labelled in the legend. Pathways (nodes) are coloured according to the functional group they belong. Nodes with no overlapping are not represented.

To further assess microbial functionality in relation to *Blastocystis* we analysed the metabolic profiles of plasma and faeces by ^1^H-NMR and HPLC-ESI-MS/MS. Then, we applied a machine learning variable selection strategy based on multiple-random forests to identify metabolites associated with the *Blastocystis* subtypes in the IRONMET discovery cohort (Fig. 6a–g). As we found the most consistent findings with ST1-3, we decided to focus in these three *Blastocystis* subtypes for the metabolomics analyses. In agreement with our previous findings, *Blastocystis-*associated shifts in the microbial functionality translated into alterations in microbial-derived metabolites from tryptophan (Tryptophan; 3-indoleacetaldehyd: 3-IAAld; 5-Hydroxyindole acetic acid: 5-HIAA; nicotinic acid, quinic acid), tyrosine (Tyrosine; 4-hydroxyphenyllactic acid: 4-HPLA, 2-phenylpropionate) and purine and pyrimidine catabolism (adenosine, hypoxanthine, uric acid) as well as those serving as 1 C sources for the one-carbon metabolism (histidine, formate, choline). To identify relevant pathways linked to metabolites associated with *Blastocystis* subtypes, we next performed a metabolite enrichment analysis based on KEGG, Reactome and Wikipathways databases (Fig. S4). This further highlighted an over-representation of pathways involved in the one-carbon metabolism (Methionine De Novo and Salvage Pathway; glycine, serine and threonine metabolism; histidine metabolism; purine metabolism, choline catabolism, folate metabolism) and aromatic amino acids metabolism (histidine, phenylalanine, tyrosine, proline, tryptophan catabolism) (Figs. 6h and S4). Due to the inherent redundancy of pathway analysis, we collapsed redundant pathways into a single biological theme using EnrichmentMap [40]. This analysis identified clusters of pathways participating in one-carbon metabolism (glycine, serine, and threonine metabolism; trans-sulfuration pathway; MTHFR deficiency) and nucleotide metabolism (Fig. 6i). It also highlighted a cluster of pathways with important roles in the central nervous systems, including neurotransmitter metabolism, Glutamatergic synapse, and GABAergic synapse. In addition, we also identified clusters linking CNS pathways with one-carbon (synaptic vesicle cycle with trans-sulfuration pathway, disorders of folate metabolism and histidine metabolism; and NT release cycle, Neuronal system and transmission across chemical synapses with choline metabolism, methionine salvage pathway, and sulfur amino acid metabolism) and aromatic amino acid pathways (phenylalanine, tyrosine and tryptophan biosynthesis and tryptophan metabolism with neurotransmitters disorders and serotonergic synapse).

In the search of an additional possible mechanisms, we took advantage of gene expression analysis of *Bifidobacterium longum* (cultured in the presence of *Blastocystis*) showing upregulation of some of the bacterium’s oxidoreductase genes, suggesting this bacterium undergoes oxidative stress in the presence of *Blastocystis* [14]. We found positive associations between serum ferritin (an oxidative stress marker) and *clr*-transformed *Blastocystis* levels both in the discovery (*Blastocystis* ST1: *r* = 0.15, *p* = 0.1; *Blastocystis* ST4: *r* = 0.18, *p* = 0.05) and validation cohorts (*Blastocystis* sp.: *r* = 0.08, *p* = 0.02). In addition, the presence of *Blastocystis* could be associated with disease not only just directly but also through reduction of beneficial bacteria [14]. We found strong negative associations between *Blastocystis* and Bifidobacteria.

### Donor’s *Blastocystis* subtypes alter recipient’s mice cognitive function and prefrontal cortex gene expression

**Fig. 7:**
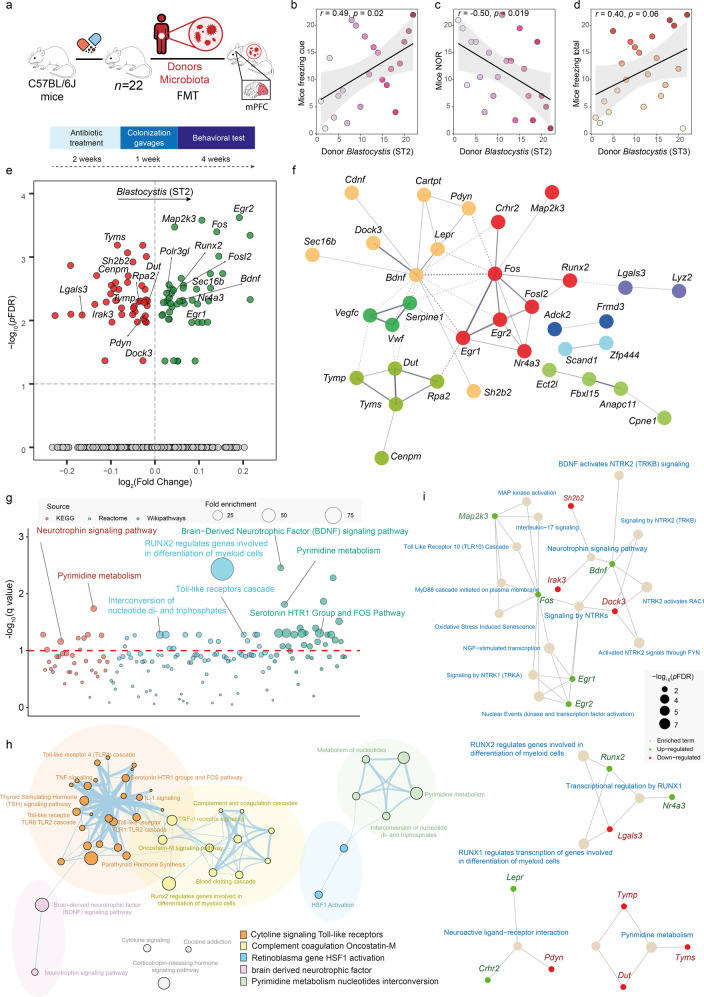
Human donor cognitive traits seem to be transmitted to recipient mice in parallel to changes in the expression of mPFC gene involved in memory formation and pyrimidine metabolism. **a** Experimental design of the faecal microbiota transplantation study. Microbiota from *n* = 22 human donors was delivered to *n* = 22 recipient mice pre-treated with antibiotics for 14 days. A freezing test and a novel object recognition test (NOR) was performed after 4 weeks. **b** Scatter plots of the Spearman’s rank correlations between the donors centered log-ratio *clr-*transformed *Blastocystis* subtypes values and the recipient mice Freezing Cue, **c** NOR, and **d** Freezing total. The ranked data is plotted. **e** Volcano plot of differentially expressed gene transcripts in the medial prefrontal cortex of the recipient mice associated with the donors *clr*-transformed *Blastocystis* ST2 values identified by limma-voom controlling for donors age, BMI, sex, and education years. The log2 fold change associated with a unit change in the *clr*-transformed values and the log10 *p* values adjusted for multiple testing (pFDR) are plotted for each transcript. Significantly different transcripts are coloured in green (upregulated) or red (down-regulated). **f** Gene interaction network constructed using differentially expressed mPFC gene transcripts associated with donors *Blastocystis* ST2 via the Search Tool for the Retrieval of Interacting Proteins/Genes (STRING) database. The network nodes are genes and the edges represent the predicted functional interactions. The thickness indicates the degree of confidence prediction of the interaction. Functional gene clusters are coloured based on the Markov Cluster Algorithm with an MCL inflation parameter of 3. Only connected nodes are shown. **g** Pathway over-representation analysis of recipient mice gene transcripts associated with the donors *clr*-transformed *Blastocystis* ST2 levels using gene sets from the KEGG, Reactome, and Wikipathways databases. The bubble size represents the fold enrichment. **h** Functional network of significant pathways obtained using Cytoscape and enrichment map. Nodes represent over-represented pathways. Node size reflects the total number of genes in each pathway. Edge thickness represents the degree of overlapping genes between pathways. Groups of functionally related pathways are labelled in the legend. Pathways (nodes) are coloured according to the functional group they belong. **i** Gene-concept network depicting significant genes involved in selected enriched pathways with important roles in the central nervous system identified using active subnetworks of interconnected genes by pathfindR.

To evaluate the potential role of *Blastocystis* subtype in cognitive function, we performed a faecal microbiota transplantation (FMT) experiment. Microbiota from 22 human donors was delivered to 22 mice after antibiotics treatment (Fig. 7a). After 4 weeks, we assessed mice cognition using the novel object recognition (NOR) and the fear conditioning tests. Recipient mice receiving microbiota from donor’s with higher *Blastocystis* ST2 levels had higher freezing cue times but lower short-time memory assessed by the NOR tests (Fig. 7b, c). Similarly, we found a positive correlation between donor’s *Blastocystis* ST3 and recipient’s mice freezing total time (Fig. 7d). In the case of *Blastocystis* ST1, we found a trend towards a negative correlation with the NOR test (*r* = −0.38, *p* = 0.085). Unlike ST1-3, we did not find significant associations between *Blastocystis* ST4 and microbial functions involved in executive function such as those participating in the one-carbon folate-mediated pyrimidine biosynthesis. In agreement with the findings in humans, we did not observe significant associations between donor’s *Blastocystis* ST4 and the cognitive tests of recipient mice. We also performed an RNA-sequencing of recipient’s mice medial prefrontal cortex (mPFC), which is implicated in executive function and memory, to identify whether donor’s *Blastocystis* subtypes could impact on mice brain transcriptome. We identified 43 down-regulated and 46 upregulated genes (out of 15,565) in the recipient’s mice mPFC associated with donor’s *Blastocystis* ST2 (Fig. 7e, Table S33). To gain better insights in the potential mechanisms underlying the *Blastocystis* ST2 on cognition, significant genes were used to build a gene-gene interaction network based on the STRING database [41]. We identified a cluster consisting mainly of upregulated immediate-early genes with well-known roles in memory formation (*Fos, Fosl2, Erg1, Erg2, Nr4a3*) (Fig. 7f). We also identified a cluster of down-regulated genes involving thymidylate synthase (*tyms*), deoxyuridine triphosphatase (*dut*) and thymidine phosphorylase (*tymp*). The bacterial functions participating in folate-mediated one-carbon metabolism thymidylate synthase (*thyX, thyA, DHFR-TS*) and *dut* were two of the microbial functions most associated with *Blastocystis* subtypes both in the IRONMET and IMAGEOMICS cohorts (Figs. 3g and 5d). To facilitate interpretation, we further analysed these results performing a pathway over-representation analysis using KEGG, Reactome, and Wikipathways databases (Fig. 7g, Fig. S5a–c) and clustering significant redundant pathways into a single term (Fig. 7h). As expected, we identified a cluster of pathways related to nucleotide metabolism (*tyms, tymp, dut, polr3gl*). Other significant clusters included pathways included the brain-derived neurotrophic factor (BDNF) signalling (*fos, egr2, dock3, egr1, sh2b2, bdnf*), the Toll-like receptor cascade (*map2k3, irak3, fos*), cytokine signalling (*fos, gbp3, egr1, map2k3, tnfrsf25, irak3*) or the regulation of genes involved in differentiation of myeloid cells by *RUNX2* (*runx2, lgals3*). Finally, to enhance determination of mechanisms underlying *Blastocystis* ST2 associations with cognition, we integrated the information provided from differential expression analysis, gene-gene interaction networks, and pathway over-representation analysis using pathfindR. First, significant genes were mapped onto a STRING gene-gene interaction network. Then, active subnetworks of interconnected genes (including genes that are not significant themselves but connect significant genes) in this gene-gene interaction network were identified. Finally, separate pathway over-representation analyses based on Reactome (Fig. S5d) and KEGG (Fig. S5e) databases were performed for each active subnetwork using the significant genes in each of the active subnetworks. At the Reactome level, this active-subnetwork-oriented pathway enrichment analysis further identified the signalling by neurotrophic-tropomyosin receptor tyrosine kinases (NTRKs) (Fig. 7i) and the RUNX1 transcription regulation of genes involved in differentiation of myeloid cells as significantly associated with *Blastocystis* ST2. At the KEGG level, it also identified the neuroactive ligand-receptor interaction (Fig. 7j).

**Fig. 8:**
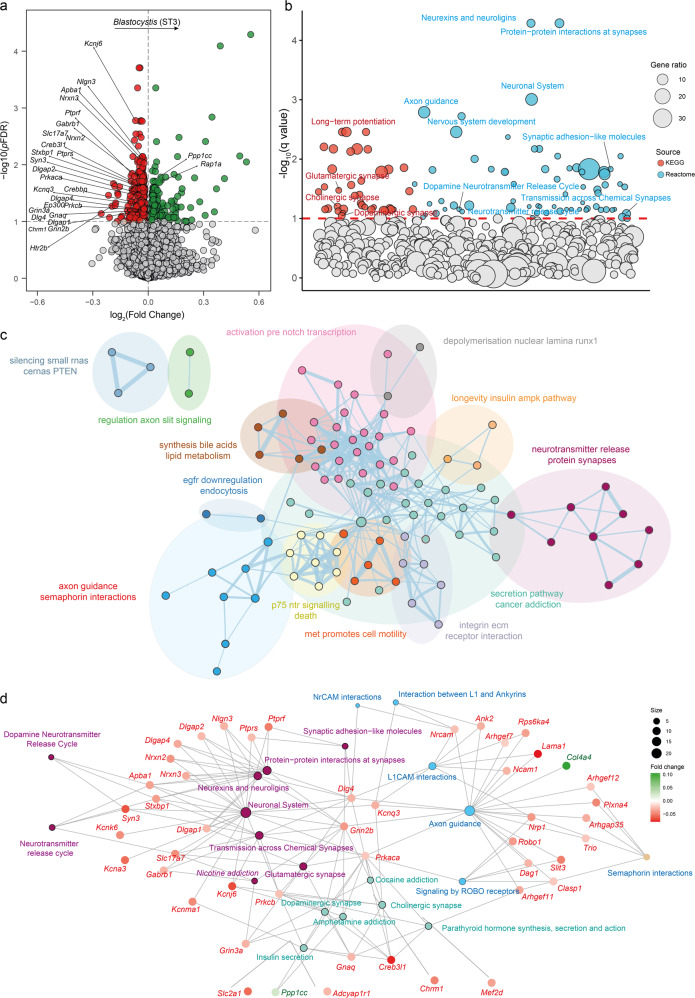
Changes in the expression of mice mPFC gene transcripts and pathways associated with *Blastocystis* ST3. **a** Volcano plot of differentially expressed gene transcripts in the medial prefrontal cortex of the recipient mice associated with the donors clr-transformed *Blastocystis* ST3 values identified by limma-voom controlling for donors age, BMI, sex, and education years. The log2 fold change associated with a unit change in the *clr*-transformed values and the log10 *p* values adjusted for multiple testing (pFDR) are plotted for each transcript. Significantly different transcripts are coloured in green (upregulated) or red (downregulated). **b** Pathway over-representation analysis of recipient mice gene transcripts associated with the donors *clr*-transformed *Blastocystis* ST3 levels based on KEGG and Reactome databases. The bubble size represents the gene ratio. **c** Functional network of significant pathways obtained using Cytoscape and enrichment map. Nodes represent over-represented pathways. Node size reflects the total number of genes in each pathway. Edge thickness represents the degree of overlapping genes between pathways. Groups of functionally related pathways are circled and labelled. Pathways (nodes) are coloured according to the functional group they belong. Nodes with no overlapping are not represented. **d** Gene-concept network depicting the linkage of significant gene transcripts involved in pathways with important roles in the central nervous system.

For donor’s *Blastocystis* ST3, we identified 248 down-regulated and 91 upregulated gene transcripts in the recipient’s mice mPFC (Fig. 8a, Table S34). A KEGG and Reactome-based pathway enrichment analysis identified an over-representation of pathways with important roles in the CNS, including glutamatergic, cholinergic, and dopaminergic synapse, neurexins and neuroligins, protein-protein interactions at synapses, and neurotransmitter release cycle (Figs. 8b and S6). After clustering redundant terms, we identified 3 clusters of pathways with important functions in the CNS, such as neurotransmitter release and protein synapses; axon guidance and L1CAM and NrCAM interactions; and dopaminergic and cholinergic synapse (Fig. 8c, d). The vast majority of genes involved in the pathways belonging to these clusters were upregulated in the mPFC of recipient’s mice with increased donor’s *Blastocystis* ST3 (Fig. 8d).

## Discussion

*Blastocystis* is a common gut eukaryote frequently detected in humans capable of asymptomatic long-term host colonisation [7, 8]. We here describe several novel findings. *Blastocystis* subtypes in the gut microbiota were associated with deficits in executive function and altered gut bacterial composition in different cohorts.

In a recent European population-level analysis of *Blastocystis* subtypes prevalence and variation in the gut microbiome of 616 healthy individuals, the researchers found that out of 69 gut microbiota covariates, only age was associated with *Blastocystis* subtype carrier status [10]. In our study we found that in the validation cohort (*n* = 942), *Blastocystis* subtype 1 levels were associated with executive functioning both in subjects ≥65 years and <60 years, whereas subtypes ST2, ST3, and ST4 were only associated with impaired executive function in subjects <60 years. In this previous study, *Blastocystis* presence was also associated with higher observed alpha diversity, with the highest diversity observed in the ST2 and ST4 samples compared to ST3 [10]. In our study, we have also found an increase in alpha diversity with higher levels of ST1 in both the discovery and validation cohorts. However, we found that higher levels of ST2 and ST3 were associated with a lower alpha diversity in the discovery cohort. In addition, we also found that both highest and lowest ST4 tertile levels were associated with higher alpha diversity compared to the middle tertile. This trend was consistently observed for ST2, ST3, and ST4 also in the validation cohort. This difference between ST1 and ST2-ST4 could explain the fact that ST1 had the strongest associations with executive function in the validation cohort. A higher alpha diversity is generally observed in healthy individuals. Therefore, the negative associations with ST1 and the other subtypes warrant further investigation.

A strong association between microbiota community composition and *Blastocystis* subtypes was also previously observed in an healthy individuals from the Flemish Gut Flora Project [10]. In line with these results, we found a strong association among all *Blastocystis* subtypes and both microbial composition and functionality. We found a strong consistent negative association among several *Bifidobacterium* and *Lactobacillus* species and the *Blastocystis* subtypes in both the validation and discovery cohorts. Consistent with our results, two recent studies have found that *Blastocystis* decreased the abundance of the beneficial bacteria *Bifidobacterium* and *Lactobacillus* [13, 14]*. Bifidobacterium breve A1* has recently shown to improve cognitive function in physically healthy older adults with suspected mild cognitive impairment [42]. *Lactobacillus plantarum* NK151 and *Bifidobacterium longum* NK173 have shown to alleviate LPS-mediated cognitive impairment in mice [43]. Similarly, *Lactobacillus casei* LC122 and *Bifidobacterium longum* BL986 improved cognitive ability in aged mice [44]. Finally, supplementation of *Bifidobacterium longum* 1714 has also shown to improve memory in healthy volunteers [45]. In addition, other genera that we identified associated with the different *Blastocystis* subtypes have also been associated with cognitive impairment in a recent study [46]. For instance, *Parabacteroides*, which we found negatively associated with *Blastocystis* subtypes, were linked to improved executive function assessed both with the backward digit span and semantic fluency span. Conversely, *Fusobacterium*, which we found positively associated with *Blastocystis* subtypes, were negatively correlated with the backward digit span [46].

Regarding the negative associations between *Blastocystis* and Bifidobacteria, past studies reported that the presence of *Bifidobacterium* attenuated both the decrease in transepithelial electrical resistance and the increased paracellular permeability of Caco-2 cells treated with LPS. *Bifidobacterium* also upregulated the expression of tight junction proteins occludin, claudin-3, and zonulin (ZO-1) and exerted anti-inflammatory properties, reducing the production of pro-inflammatory cytokines IL-6 and TNF-α [47]. In contrast, *Blastocystis* ST7 disrupted tight junction proteins such as occludin and ZO-1 [48, 49] as well as increases the levels of pro-inflammatory cytokines to trigger an inflammatory response [50, 51]. In addition, *Lactobacillus rhamnosus* and *Bifidobacterium longum* have shown to alleviate cognitive impairment in mice by regulation the IFN-γ/IL-10 and TNF-α/IL-10 rations [52], whereas *Lactobacillus plantarum* and *Bifidobacterium longum* have also shown to alleviate cognitive impairment in mice by up-regulating NF-kB-mediated BDNF expression [43]. A recent study has shown that *Bifidobacterium breve* prevented memory impairment a mouse model with phenotypic features of Alzheimer’s disease through microglia activation and the reduction of amyloid-β production [53].

We also observed inter-relationships between bacterial function, specifically those related to pyrimidine, one-carbon, and aromatic amino acids metabolism and *Blastocystis* abundance. In particular, ST1, ST2 and ST3 were strongly associated with thymidylate synthase (*thyX, thyA)* and dUTP pyrophosphatase (*dut*), both involved in the folate-mediated one-carbon metabolism, which we have previously shown to play a critical role in executive function [5]. The *thyX* gene encodes for thymidylate synthase (*TYMS* in humans). Decreased *TYMS* expression levels lead to imbalances between DNA synthesis and methylation, which is essential for neurodevelopment, synaptic plasticity and memory [54]. Impairments in folate-mediated one-carbon metabolism have been associated with neurodegenerative disease that may result from dTMP synthesis impairment and consequent uracil misincorporation into DNA [55, 56]. These *Blastocystis*-associated shifts in bacterial functionality translated into the circulating metabolome. Finally, donor’s *Blastocystis* subtypes led to altered recipient’s mice cognitive function and prefrontal cortex gene expression. All these findings support that the proposed commensal role for Blastocystis should be viewed with caution.

Several potential pathophysiologic factors linked to the relationships of *Blastocystis* with cognition should also be taken into account. Some studies on non-transformed rat intestinal epithelial cells showed that both live parasites and lysate of ST4 increased intestinal permeability [57]. Parasite cysteine proteases resulted in loss of zonulin protein and F-actin reorganization [49]. Loss of zonulin and occludin protein was found to correlate to subtype with ST7 resulting in increased permeability compared to negligible effects of ST4 in human intestinal epithelium.

The potentially induced increased permeability could have contributed to the differential metabolome observed: Alterations in microbial-derived metabolites from tryptophan (Tryptophan; 3-indoleacetaldehyd: 3-IAAld; 5-Hydroxyindole acetic acid: 5-HIAA; nicotinic acid, quinic acid), tyrosine (Tyrosine; 4-hydroxyphenyllactic acid: 4-HPLA, 2-phenylpropionate) and purine and pyrimidine catabolism (adenosine, hypoxanthine, uric acid) as well as those serving as 1C sources for the one-carbon metabolism (histidine, formate, choline).

In addition, *Blastocystis* seem to cause mucosal sloughing, increase in goblet cell mucin, and to induce a pro-inflammatory cytokine response with local upregulation of TNF-α, IFN-γ and IL-12 [58]. The systemic levels of these cytokines are implicated in the development of cognitive dysfunction [59]. Therefore, the possible impact of the presence of *Blastocystis* in the gut on cognition through these mechanisms should be further investigated.

In summary, the evidence found in the discovery and replication cohorts and the transmission of cognitive traits through microbiota transplantation suggest that the presence of *Blastocystis* in the gut microbiota may constitute a marker for a poor cognition. This was especially remarkable in subjects aged 50 to 65 years while the observations were not consistent in subjects above this age, possibly due to a survival effect, as seen with other cardiovascular risk factors.

## Supplementary information

Supplementary Figures

Supplementary Methods

## Data Availability

The primary database are publicly available at the repository DataDryad at the following url: https://datadryad.org/stash/share/LZYWcISzIH8kA5tZLA8_710XzeBNj1096uVk_rw5WRM. The raw metagenomic sequence data of the 114 human subjects from the Ironmet cohort have been deposited in the European Nucleotide Archive (ENA) under the project number PRJEB39631 with the accession numbers ERS4859818-ERS4859933. The raw metagenomic sequence data of the 942 human subjects from the Imageomics cohort have been deposited in the European Nucleotide Archive (ENA) under the project number PRJEB52682 with the accession numbers ERA13584434.
