# Supplementary material for: Presence of Blastocystis in gut microbiota is associated with cognitive traits and decreased executive function
Source: ISME J. 2022 Jun 21;16(9):2181–97. doi: 10.1038/s41396-022-01262-3 (PMC9381544; doi:10.1038/s41396-022-01262-3)
Supplement: Supplementary file 1 — Supplementary Figures [file 41396_2022_1262_MOESM1_ESM.pdf]

Supplementary Figure 1

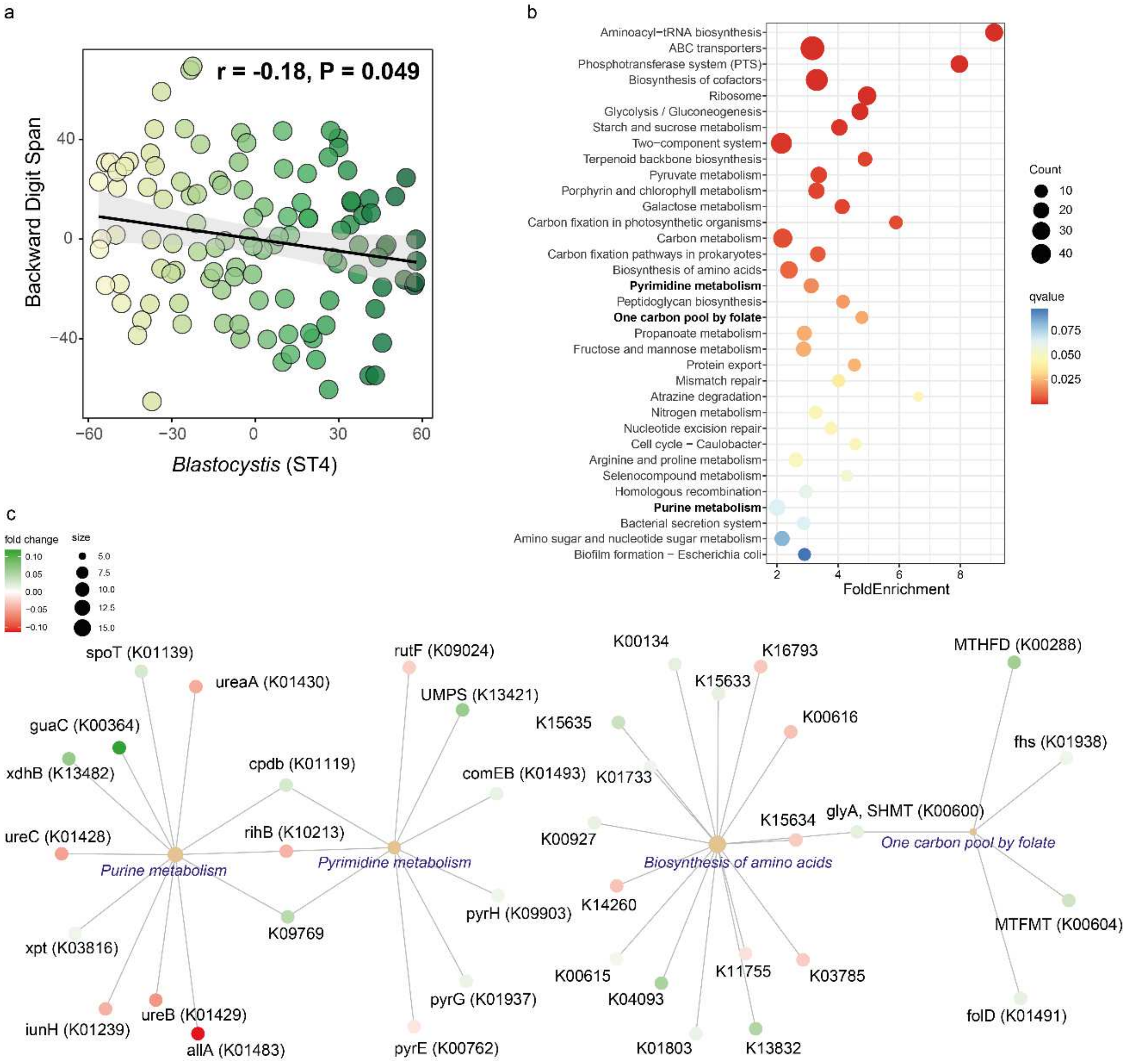

Figure S1. *Blastocystis* subtype 4 is moderately associated with executive function and bacterial functionality. a) Scatter plots of the partial Spearman's rank correlations (adjusted for age, BMI, sex and education years) between the baseline faecal centered log-ratio *clr*-transformed *Blastocystis* subtype 4 (ST4) values and executive function assessed by the backward digit span tests (IRONMET, *n*=114) at baseline. The ranked residuals are plotted. **b)** Dotplot of the KEGG pathway over-representation analysis (*q*-value<0.1) mapping the KEGG orthologues significantly associated with ST4. Dots are coloured according to the *q*value. **c)** Gene-concept network depicting the linkage of significant KEGG orthologues associated with ST4 participating in KEGG pathways related to the purine and pyrimidine metabolism, one-carbon metabolism, and biosynthesis of amino acids.

Supplementary Figure 2

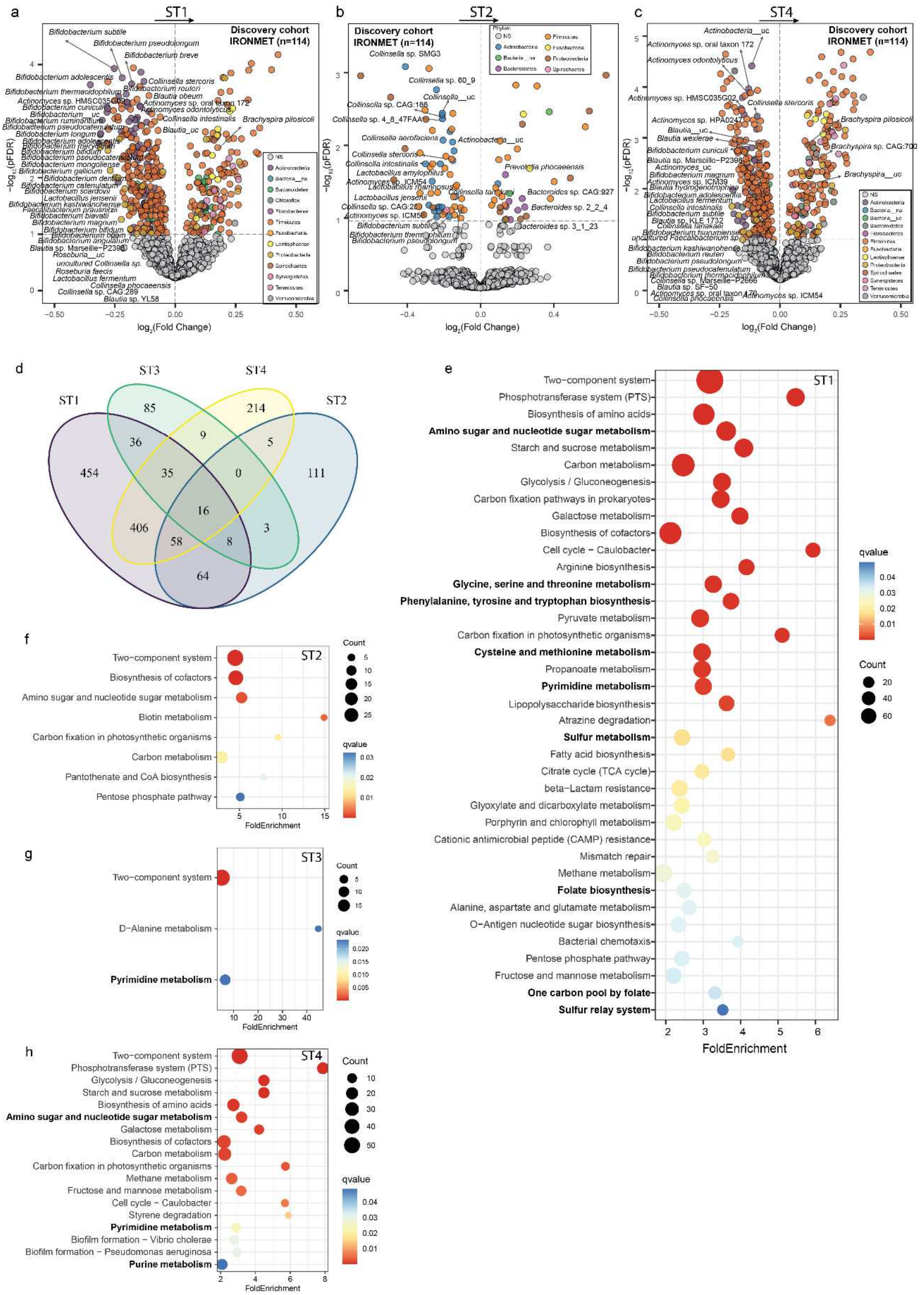

Figure S2. Associations of *Blastocystis* subtypes with the clr-transformed bacterial composition and functionality in the discovery cohort. **a)** Volcano plots of bacterial taxa associated with the faecal clr-transformed *Blastocystis* subtype 1 (ST1), **b)** subtype 2 (ST2), and **c)** subtype 4 (ST4) values identified applying linear models to the *clr*-transformed bacterial data controlling for age, sex, BMI, and education years in the discovery cohort (IRONMET, *n*=114). The log2 fold change associated with a unit change in the *clr*-transformed values and the log10 *p*values adjusted for multiple testing (pFDR) are plotted for each taxon. Significantly different taxa are coloured according to phylum. **d)** Dot plots of the KEGG pathway over-representation analyses (*q*value<0.1) mapping the KEGG orthologues significantly associated (identified using the clr-transformed ortholog data) with ST1, **e)** ST2, **f)** ST3, and **g)** ST4 in the discovery cohort (IRONMET, *n*=114). Dots are coloured according to the *q*value. **h)** Venn diagram representing the overlap of *clr*-transformed KEGG orthologues significantly associated with the different *Blastocystis* subtypes in the discovery cohort (IRONMET, *n*=114).

Supplementary Figure 3

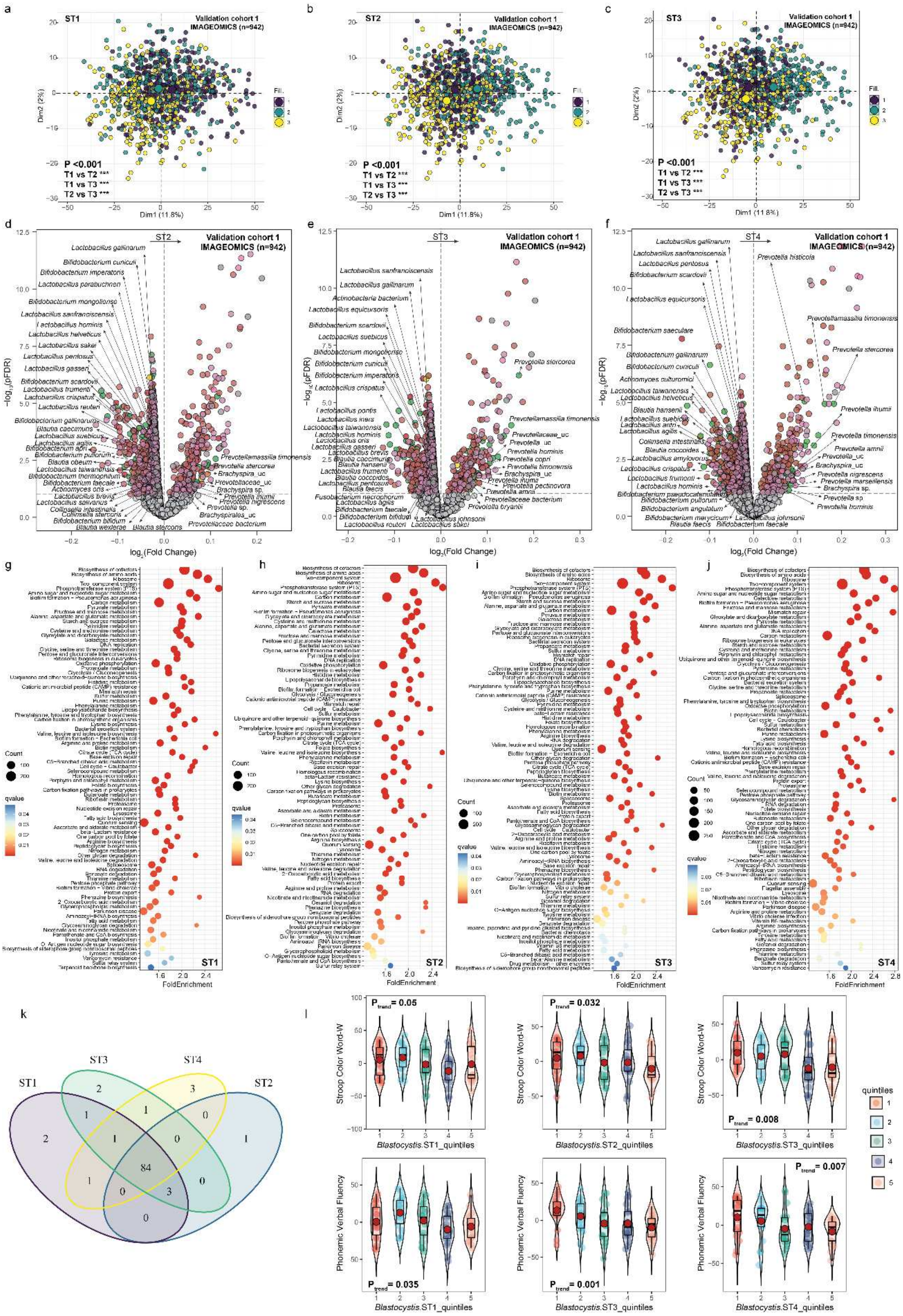

Figure S3. Associations of *Blastocystis* subtypes with the clr-transformed bacterial composition and functionality in the validation cohort. a) Volcano plots of bacterial taxa associated with the faecal clr-transformed *Blastocystis* subtype 2 (ST2), **b)** subtype 3 (ST3), and **c)** subtype 4 (ST4) values identified applying linear models to the clr-transformed bacterial data controlling for age, sex, BMI, and education years in the validation cohort (IMAGEOMICS, *n*=942). The log2 fold change associated with a unit change in the *clr*-transformed values and the log10 *p*values adjusted for multiple testing (pFDR) are plotted for each taxon. Significantly different taxa are coloured according to phylum. **d)** Dot plots of the KEGG pathway over-representation analyses (*q*value<0.1) mapping the KEGG orthologues significantly associated (identified using the clr-transformed ortholog data) with ST1, **e)** ST2, **f)** ST3, and **g)** ST4 in the validation cohort (IMAGEOMICS, *n*=942). Dots are coloured according to the *q*value. **h)** Venn diagram representing the overlap KEGG pathways significantly associated with the different *Blastocystis* subtypes in the validation cohort (IMAGEOMICS, *n*=942).

Supplementary Figure 4

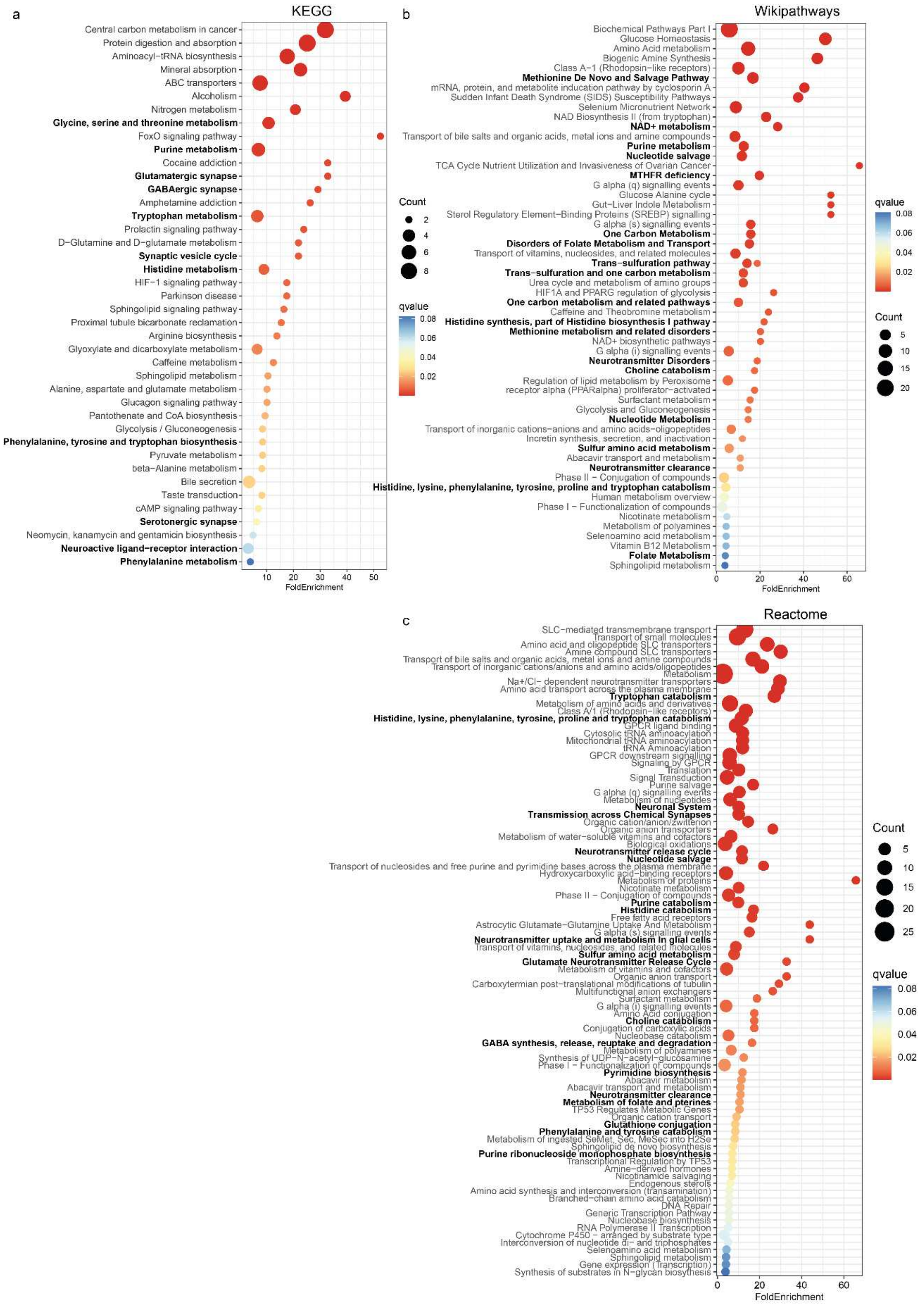

Figure S4. Over-representation analysis of metabolites associated with the *Blastocystis* subtypes. a) Dot plot of the pathway over-representation analyses (*q*value<0.1) mapping the significant plasma and faecal metabolites associated the *clr*-transformed *Blastocystis* ST1-3 to the KEGG, **b)** Wikipathways and **c)** Reactome databases. Dot size is proportional to the number of metabolites in each pathway and dots are coloured according to the *q*value.

Supplementary Figure 5

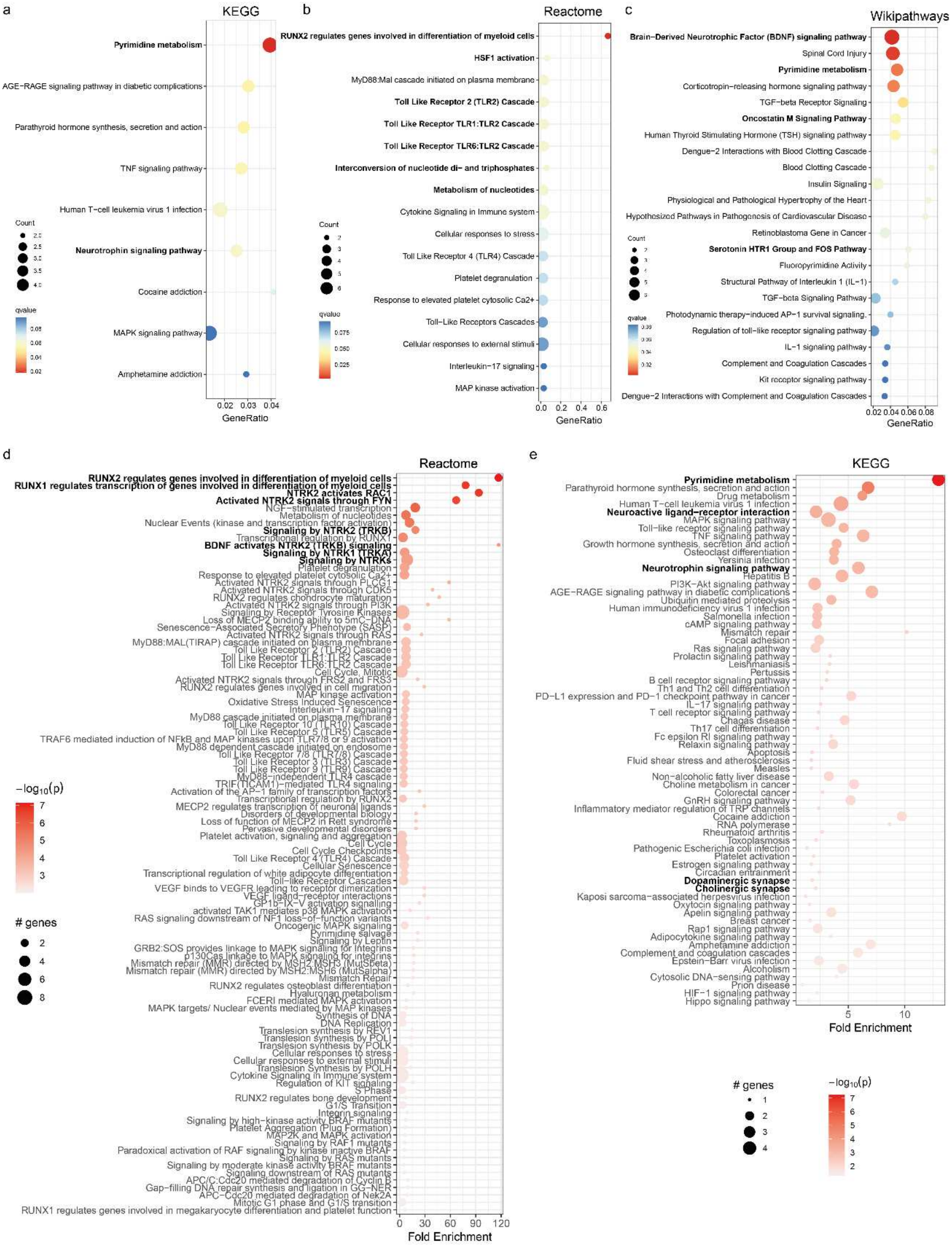

Figure S5. Over-representation analysis of recipient mice mPFC gene transcripts associated with the *Blastocystis* ST2. a) Dotplots of the pathway over-representation analyses (*q*value<0.1) mapping the significant recipient mice gene transcripts associated with the clr-transformed *Blastocystis* ST3 to the KEGG, **b)** Reactome and **c)** Wikipathways databases. **d**) Dotplot of over-represented pathways identified using active subnetworks of interconnected genes with pathfindR using gene sets from the Reactome and **e)** KEGG databases. Dot size is proportional to the number of gene transcripts in each pathway and dots are coloured according to the *q*value.

Supplementary Figure 6

a

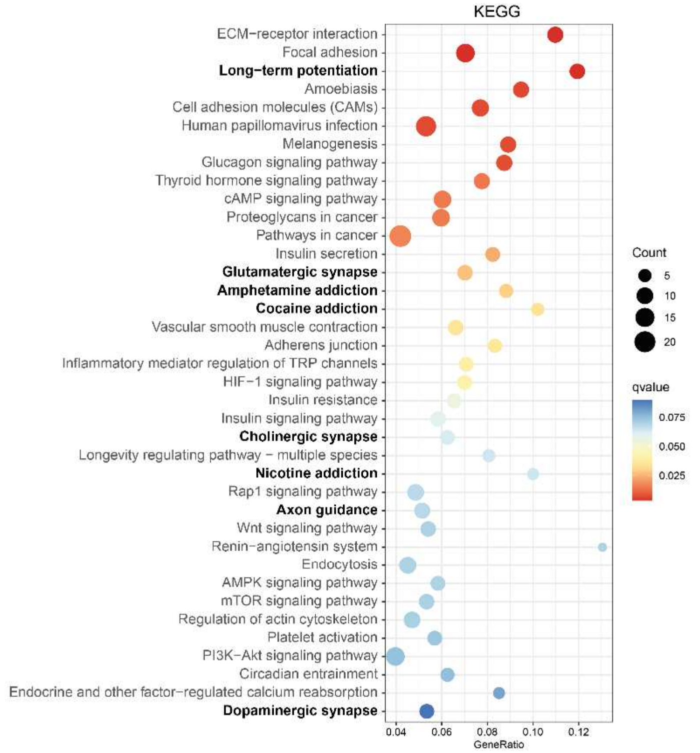

b

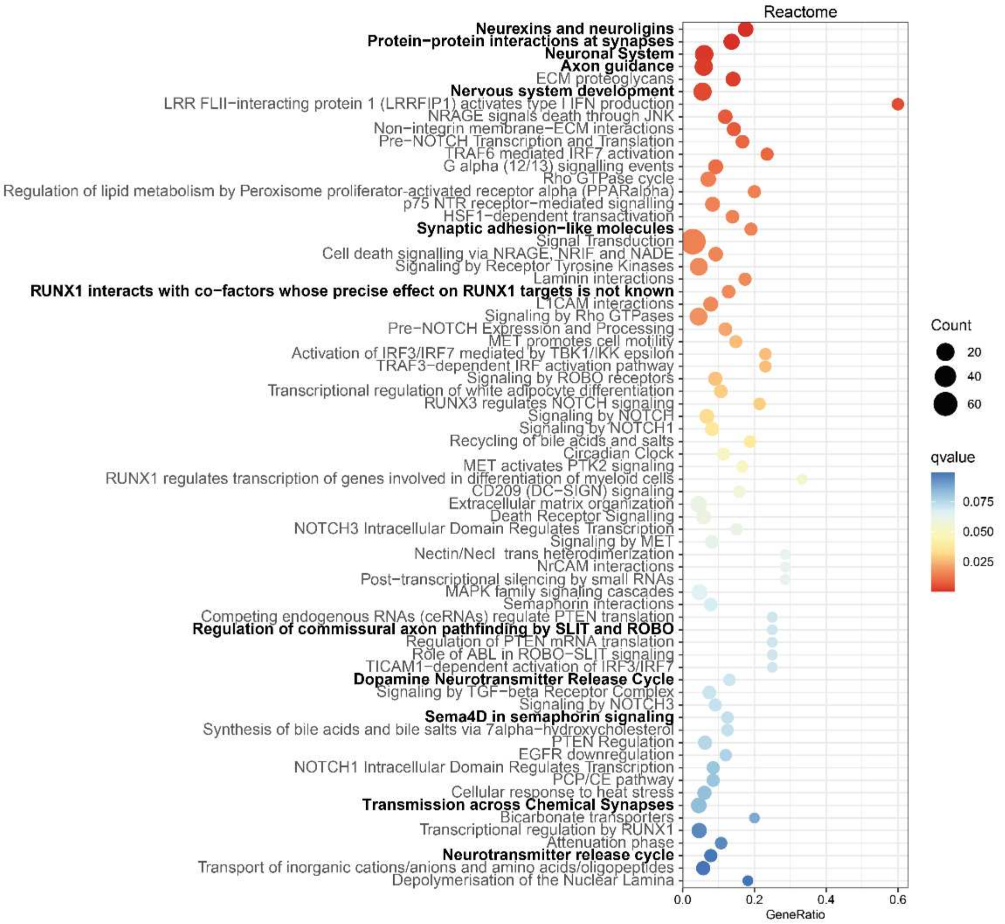

Figure S6. Over-representation analysis of recipient mice mPFC gene transcripts associated with the *Blastocystis* ST3. a) Dotplots of the pathway over-representation analyses (*q*value<0.1) mapping the significant recipient mice gene transcripts associated with the clr-transformed *Blastocystis* ST3 to the gene sets from the KEGG and **b)** Reactome databases. Dot size is proportional to the number of gene transcripts in each pathway and dots are coloured according to the *q*value.
