# Supplementary material for: Presence of Blastocystis in gut microbiota is associated with cognitive traits and decreased executive function
Source: ISME J. 2022 Jun 21;16(9):2181–97. doi: 10.1038/s41396-022-01262-3 (PMC9381544; doi:10.1038/s41396-022-01262-3)
Supplement: Supplementary file 2 — Supplementary Methods [file 41396_2022_1262_MOESM2_ESM.docx]

**Metabolomics Analyses**

*^1^H-Nuclear Magnetic Resonance (IRONMET)*

From faecal samples, 15-20 mg of dried faecal matter was placed in a 2 ml Eppendorf tube. Then, 500 µL of 0.05 M PBS buffer in H_2_O (pH=7.3) was added and vortexed vigorously, frozen and thawed twice and centrifuged (21000 g, 15 min, 4°C) to obtain a clear faecal water over the precipitated stool. From the upper layer, 200 µL of prepared faecal water was placed in appropriate 2 ml Eppendorf tube and then, 400 µL of 0.05M PBS buffer in D_2_O (pH=7.2, TSP 0.7mM) was added. The sample was vigorously vortexed and sonicated until complete homogenization and the mixture (clear dispersion), if necessary, was centrifuged again (14000 rpm around 14000 g, 5 min, 4ºC). For NMR measurement the clear upper phase was placed into a 5mm o.d. NMR tube. One dimensional ^1^H pulse experiments were carried out using the NOESY-presaturation sequence [recycle delay (RD)-90º–1–90º–m–90º acquire (ACQ) free induction decay (FID)] to suppress the residual water peak. For each sample, 8 dummy scans were followed by 256 scans and collected in 64-K points over a spectral width of 20 ppm. All ^1^H-NMR spectra were recorded at 300 K on an Avance III 600 spectrometer (Bruker®, Germany) operating at a proton frequency of 600.20 MHz using a 5 mm PABBO gradient probe and automatic sample changer with a cooling rack at 4ºC.

*HPCL-ESI-MS/MS metabolomics analyses (IRONMET).*

Metabolites were extracted from plasma and faecal samples with methanol (containing phenylalanine-C13 as an internal standard) according to previously described methods (1). Briefly, for plasma samples 30µl of cold methanol were added to 10 µl of each sample, vortexed for 1 minute and incubated for one hour at −20 °C. For faecal samples, the content of a 1.2 ml tube of Lysing Matrix E (MP biomedicals) and 600 μl of cold methanol were added to 10mg of sample. Samples were homogenized using FastPrep-24™ (MP biomedicals) and were incubated overnight in a rocker at 4°C. Then, all samples were centrifuged for three minutes at 12.000g, the supernatant was recovered and filtered with a 0.2 μm Eppendorf filter. Two µL of the extracted sample were applied onto a reversed-phase column (Zorbax SB-Aq 1.8 µm 2.1 x 50 mm; Agilent Technologies) equipped with a precolumn (Zorbax-SB-C8 Rapid Resolution Cartridge 2.1 x 30 mm 3.5 µm; Agilent Technologies) with a column temperature of 60°C. The flow rate was 0.6 mL/min. Solvent A was composed of water containing 0.2% acetic acid and solvent B was composed of methanol 0.2% acetic acid. The gradient started at 2% B and increased to 98% B in 13 min and held at 98% B for 6 min. Post-time was established in 5 min.

Data were collected in positive and negative electrospray modes in a Q-TOF 6520 operated in full-scan mode at 50–3000 m/z in an extended dynamic range (2 GHz), using N2 as the nebulizer gas (5 L/min, 350°C). The capillary voltage was 3500 V with a scan rate of 1 scan/s. The ESI source used a separate nebulizer for the continuous, low-level (10 L/min) introduction of reference mass compounds 121.050873 and 922.009798, which were used for continuous, online mass calibration. MassHunter Data Analysis Software (Agilent Technologies, Barcelona, Spain) was used to collect the results, and MassHunter Qualitative Analysis Software (Agilent Technologies, Barcelona, Spain) to obtain the molecular features of the samples, representing different, co-migrating ionic species of a given molecular entity using the Molecular Feature Extractor algorithm (Agilent Technologies, Barcelona, Spain). We selected samples with a minimum of 2 ions. Multiple charge states were forbidden. Compounds from different samples were aligned using a retention time window of 0.1% ± 0.25 minutes and a mass window of 20.0 ppm ±2.0 mDa. We selected only those present in at least 50% of the samples of one group and corrected for individual bias.

**Faecal Microbiota Transplantation study**

*Animals*

Male C57BL/6J mice (Charles River, France), weighing 23–26 g at the beginning of the experiment were used in this study. Mice were housed individually in controlled laboratory conditions with the temperature maintained at 21 ± 1ºC, humidity at 55 ± 10%, and 7h30/19h30 light/dark cycles. All animals were fed a standard chow diet RM1 (Irradiated Vacuum packed, Dietex International Ltd.). The health status of each mouse included in the experimental schedule was checked every day before the experimental sessions and recorded in the experimenter protocol notebook. Health status checks included body weight, physical aspect, behaviour, and clinical signs. No abnormalities were recorded in the animals included in this study. Animal procedures were conducted in strict accordance with the guidelines of the European Communities Directive 86/609/EEC regulating animal research and were approved by the local ethical committee (CEEA-PRBB). All the experiments were performed under blinded conditions (the researcher who administered the microbiota was blinded in relation to the memory scores of the subjects who provided the faeces).

*Experimental design*

Mice were given a cocktail of ampicillin and metronidazole, vancomycin (all at 500 mg/L), ciprofloxacin HCl (200 mg/L), imipenem (250 mg/L) once daily for 14 consecutive days in drinking water, as previously described (2). Seventy-two hours later, animals were colonized via daily oral gavage of donor microbiota (150 mL) for 3 days. Animals were orally gavaged with faecal material from healthy human donors’ samples (*n*=22) matched for age, BMI, sex and education years. To offset potential confounder and/or cage effects and to reinforce the donor microbiota phenotype, booster inoculations were given twice per week throughout the study. Animals were exposed to a series of behavioural testing including novel object recognition (NOR) test and fear conditioning with nociception assessed by the hot plate test to ensure specificity. At the end of the study the animals were consecutively sacrificed.

*The novel object recognition test*

The novel object recognition (NOR) test was performed in a V-maze as previously published (3). Three phases of 9-min were performed on consecutive days. Mice were first habituated to the V-maze. On the second day, 2 identical objects (chess pieces) were presented to the mice, and the time that they spent exploring each object was recorded. In the test phase (3 h later for short-term memory or 24 h later for long-term memory), 1 of the familiar objects was replaced with a novel object (a different chess piece), and the time spent exploring each object (novel and familiar) was computed. A discrimination index was calculated as the difference between the times that the animal spent exploring the novel (Tn) and familiar (Tf) object divided by the total time of object exploration: (Tn-Tf)/(Tn + Tf).

*Fear conditioning*

Fear conditioning was conducted as described previously with some modifications (4). Mice were individually placed in a shuttle chamber (LE918, Panlab, Barcelona) surrounded by a sound-attenuating cabinet. The chamber floor was formed by parallel stainless-steel bars connected to a scrambled shock generator. On the training day, mice were habituated to the chamber during 180 s before the exposure to an acute beeping 30 s sound (80 dB). Each animal received an unconditioned stimulus (US) (0.6 mA footshock during 2 s) paired with the end of the sound (conditioned stimulus, CS). After the shock, the animal remained for 60 s in the shuttle chamber. To evaluate cued fear conditioning, mice were re-exposed to the CS in a novel environment (a wide white cylinder in the chamber) 24 h after the conditioning session. Mice were allowed to adapt for 180 s to the new environment which was followed by 30 s of the sound used in the training day. After the last sound trial, mice remained in the cylinder for 60 s. Fear memory was assessed as the percentage of time that mice spent freezing during the session. Freezing response, a rodent’s natural response to fear, was evaluated by direct observation and defined as complete lack of movement, except for respiration for more than 1 s. The procedure was performed between 8.00 and 12.00 h in an experimental room different to the housing room.

**Study of Gene Expression in mouse Prefrontal Cortex**

*Sample Preparation*

The mice brains were quickly removed and the medial prefrontal cortex was dissected according to the atlas of stereotaxic coordinates of mouse brain (5). Brain tissues were then frozen by immersion in 2-methylbutane surrounded by dry ice, and stored at -80ºC.

*RNA Quality Control*

Quality control of the RNA was performed using the RNA 6000 Nano chip (Agilent) on an Agilent Bioalyzer 2100 obtaining RIN values between 8.7 - 9.8.

*RNA Libraries*

Libraries were prepared from 500 ng of total RNA using the TruSeq stranded mRNA library preparation kit (Illumina, #20020594) with TruSeq RNA Single Indexes (Illumina, #20020492 and #20020493) according to the manufacturer’s instruction reducing the RNA fragmentation time to 4.5 min. Prepared libraries were analysed on a DNA 1000 chip on the Bioanalyzer and quantified using the KAPA Library Quantification Kit (Roche, #07960204001) on an ABI 7900HT qPCR instrument (Applied Biosystems). Sequencing was performed with 2x50 bp paired-end reads on a HiSeq 2500 (Illumina) using HiSeq v4 sequencing chemistry. Raw sequencing reads in the fastq files were mapped with STAR version 2.5.3a (6) to the Gencode release 17 based on the GRCm38.p6 reference genome and the corresponding GTF file. The table of counts was obtained with FeatureCounts function in the package subread, version 1.5.1 (7).

**Statistical analyses**

In the IRONMET discovery cohort, partial Spearman’s correlation analysis was used to determine the correlation between cognitive variables and clr-transformed *Blastocystis* subtypes after controlling for age, BMI, sex, and education years. Accordingly, scatter plots were generated with the ranked residuals of the model adjusting for selected covariates. In the IMAGEOMICS validation cohort, the ranked residuals of cognitive tests scores were plotted according to the quartiles or quintiles of the clr-transformed *Blastocystis* sp*.* levels after controlling for age, BMI, sex, and education years. Non-parametric monotonic trends according the *Blastocystis* sp*.* quartiles or quintiles were assessed by the Mann-Kendall trend test.

*Metagenomics statistical analysis*

To take into account the compositional structure of the microbiome data and rule out possible spurious associations, we applied a centred log-ratio (clr) transformation to the *Blastocystis* raw counts using the “ALDEx2” R package (8). It first uses a Dirichlet-multinomial model to inter abundance from read counts and then applies a clr transformation to each instance. We used 128 Dirichlet Monte Carlo instances in the aldex.clr function. Bacterial species and functions differentially associated with the clr-transformed *Blastocystis* subtypes levels were identified using the “DESeq2” R package (9), adjusting for age, BMI, sex, and education years. To specifically account for the compositional and zero-inflated properties of the microbiome data, raw counts were normalized using the geometric mean of pairwise ratios (GMPR). (10) Taxa and bacterial functions were previously filtered so that only those with more than 10 reads in at least 10% of the samples were selected. The *p*values for bacterial taxa were then adjusted for multiple comparisons using the Benjamini-Hochberg procedure for False Discovery Rate (pFDR). For bacterial functionality, pathway over-representation analyses was performed mapping significant KEGG orthologs associated with the clr-transformed *Blastocystis* subtypes to the KEGG pathways using the “enrichKEGG” function from the “ClusterProflier” R package (11). Pathway significance was assessed using a hypergeometric test and a Storey procedure (*q*values) was applied for multiple testing correction. Gene concept networks for selected pathways were plotted using the “cnetplot” function.

*Metabolomics statistical analysis*

Metabolomics data were first normalized using a probabilistic quotient normalisation. Then data were analysed using machine learning (ML) methods. In particular, we adopted an all-relevant ML variable selection strategy applying a multiple random forest (RF)-based method as implemented in the Boruta algorithm (12). It has been recently proposed as one of the two best-performing variable selection methods making use of RF for high-dimensional omics datasets (13). The Boruta algorithm is a wrapper algorithm that performs feature selection based on the learning performance of the model (12). It performs variables selection in three steps: a) Randomization, which is based on creating a duplicate copy of the original features randomly permutate across the observations; b) Model building, based on RF with the extended data set to compute the normalized permutation variable importance (VIM) scores; c) Statistical testing, to find those relevant features with a VIM higher than the best randomly permutate variable using a Bonferroni corrected two-tailed binomial test; and d) Iteration, until the status of all features is decided. We run the Boruta algorithm with 500 iterations, a confidence level cut-off of 0.005 for the Bonferroni adjusted *p*values, 5000 trees to grow the forest (ntree), and a number of features randomly sampled at each split given by the rounded down number of features/3 (the mtry recommended for regression). Pathway over-representation analysis was performed mapping metabolites that were significantly associated with the *Blastocystis* subtypes in the discovery cohort to the KEGG, Reactome, and Wikipathways included in the ConsensusPathDB (14). Pathway significance was assessed using a hypergeometric test and a Storey procedure (*q*values) was applied for multiple testing correction. Enriched pathways were then mapped as a functional network of pathways using “Cytoscape” and the “EnrichmentMap” plugin.

*RNA-seq analysis.* Differential expression gene analyses were performed on gene counts using the “limma” R package (15). First, low expressed genes were filtered, so that only gene with more than 10 reads in at least 2 samples were selected. After filtering, 15,565 genes out of 22,204 were retained for subsequent analyses. RNA-seq data were then normalized for RNA composition using the trimmed mean of M-value (TMM) as implemented in edgeR package (16). Normalized counts were then converted to log2 count per million (logCPM) with associated precision weights to account for variations in precision between different observations using the “voom” function with donor’s age, BMI, sex, and education years as covariates. A robust linear regression model adjusted the previous covariates was then fitted to the data using the “lmFit” function with the option method = “robust”, to limit the influence of outlying samples. Finally, an empirical Bayes method was applied to borrow information between genes with the “eBayes” function. *p*values were adjusted for multiple comparisons using the Benjamini-Hochberg procedure for False Discovery Rate (pFDR). Alternatively, the Sequential Goodness of Fit (17) as implemented in the “SGoF” R package was also used. Unlike FDR methods, which decrease their statistical power as the number of test increases, SGoF methods increase their power with increasing number of tests. SGoF has proven to behave particularly better than FDR methods with high number of tests and low sample size, which is the case of omics large datasets. Differentially expressed genes were mapped to the Search Tool for Retrieval of Interacting Proteins/Genes (STRING) database (which integrates known and predicted protein/gene interactions) to predict functional gene-gene interaction networks (18). Then, functional local clusters in the interaction network were determined using a Markov Cluster algorithm (MCL) with an inflation parameter = 3. Active interacting sources including text mining, experiments, databases, co-expression, and co-occurrence and an interaction score > 0.4 were used to construct the interaction networks. In addition, the functional roles of differentially expressed genes were characterized using over-representation analyses based on the KEGG, Reactome and Wikipathways databases using ConsensusPathDB (14). Pathway significance was assessed using a hypergeometric test and a Storey procedure (*q*values) was applied for multiple testing correction. Additionally, we integrated the information provided from differential expression analysis, gene-gene interaction networks, and pathway over-representation analysis using the R package “pathfinder” (19). First, significant genes were mapped onto a STRING gene-gene interaction network. Then, active subnetworks of interconnected genes (including genes that are not significant themselves but connect significant genes) in this gene-gene interaction network were identified. Finally, separate pathway over-representation analyses based on Reactome and KEGG databases were performed for each active subnetwork using the significant genes in each of the active subnetworks.

**References**

1. Wikoff, W. R., Pendyala, G., Siuzdak, G. & Fox, H. S. Metabolomic analysis of the cerebrospinal fluid reveals changes in phospholipase expression in the CNS of SIV-infected macaques. *J. Clin. Invest.* **118**, 2661–9 (2008).
2. Kelly, J. R. *et al.* Transferring the blues: Depression-associated gut microbiota induces neurobehavioural changes in the rat. *J. Psychiatr. Res.* **82**, 109–118 (2016).
3. Burokas, A. *et al.* Relationships between serotonergic and cannabinoid system in depressive-like behavior: a PET study with [11C]-DASB. *J. Neurochem.* **130**, 126–35 (2014).
4. Saravia, R. *et al.* Concomitant THC and stress adolescent exposure induces impaired fear extinction and related neurobiological changes in adulthood. *Neuropharmacology* **144**, 345–357 (2019).
5. Paxinos, G. & Franklin, K. B. J. The mouse brain in stereotaxic coordinates. (Academic Press, 1997).
6. Dobin, A. *et al.* STAR: Ultrafast universal RNA-seq aligner. *Bioinformatics* **29**, 15–21 (2013).
7. Liao, Y., Smyth, G. K. & Shi, W. FeatureCounts: An efficient general purpose program for assigning sequence reads to genomic features. *Bioinformatics* **30**, 923–930 (2014).
8. Fernandes, A. D. *et al.* Unifying the analysis of high-throughput sequencing datasets: Characterizing RNA-seq, 16S rRNA gene sequencing and selective growth experiments by compositional data analysis. *Microbiome* **2**, 15 (2014).
9. Love, M. I., Huber, W. & Anders, S. Moderated estimation of fold change and dispersion for RNA-seq data with DESeq2. *Genome Biol.* **15**, 550 (2014).
10. Chen, L. *et al.* GMPR: A robust normalization method for zero-inflated count data with application to microbiome sequencing data. *PeerJ* **2018**, (2018).
11. Wu, T. *et al.* clusterProfiler 4.0: A universal enrichment tool for interpreting omics data. *Innov.* 100141 (2021) doi:10.1016/J.XINN.2021.100141.
12. Kursa, M. B. & Rudnicki, W. R. Feature selection with the boruta package. *J. Stat. Softw.* **36**, 1–13 (2010).
13. Degenhardt, F., Seifert, S. & Szymczak, S. Evaluation of variable selection methods for random forests and omics data sets. *Brief. Bioinform.* **20**, 492–503 (2019).
14. Kamburov, A., Stelzl, U., Lehrach, H. & Herwig, R. The ConsensusPathDB interaction database: 2013 Update. *Nucleic Acids Res.* **41**, D793–D800 (2013).
15. Ritchie, M. E. *et al.* Limma powers differential expression analyses for RNA-sequencing and microarray studies. *Nucleic Acids Res.* **43**, e47 (2015).
16. Robinson, M. D., McCarthy, D. J. & Smyth, G. K. edgeR: A Bioconductor package for differential expression analysis of digital gene expression data. *Bioinformatics* **26**, 139–140 (2010).
17. Carvajal-Rodríguez, A., de Uña-Alvarez, J. & Rolán-Alvarez, E. A new multitest correction (SGoF) that increases its statistical power when increasing the number of tests. *BMC Bioinformatics* **10**, 209 (2009).
18. Szklarczyk, D. *et al.* STRING v11: Protein-protein association networks with increased coverage, supporting functional discovery in genome-wide experimental datasets. *Nucleic Acids Res.* **47**, D607–D613 (2019).
19. E, U., O, O. & OU, S. pathfindR: An R package for comprehensive identification of enriched pathways in omics data through active subnetworks. *Front. Genet.* **10**, (2019).
